# Evaluation of Subetadex-α-methyl, a Polyanionic Cyclodextrin Scaffold, as a Medical Countermeasure against Fentanyl and Related Opioids

**DOI:** 10.1021/acscentsci.4c00682

**Published:** 2024-10-23

**Authors:** Michael A. Malfatti, Heather A. Enright, Summer McCloy, Esther A. Ubick, Edward Kuhn, Alagu Subramanian, Victoria Hio Leong Lao, Doris Lam, Nicholas A. Be, Saphon Hok, Edmond Y. Lau, Derrick C. Kaseman, Brian P. Mayer, Carlos A. Valdez

**Affiliations:** ^†^Physical and Life Sciences Directorate, ^‡^Biosciences and Biotechnology Division, ^§^Global Security Directorate, ^∥^Forensic Science Center, and ^⊥^Materials Science Division, Lawrence Livermore National Laboratory, Livermore, California 94550, United States

## Abstract

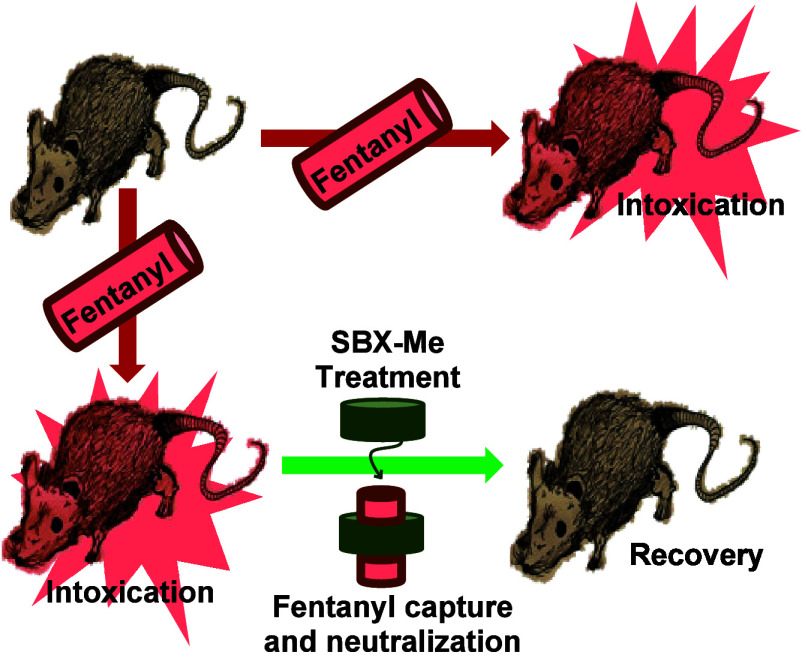

Subetadex-α-methyl (SBX-Me), a modified, polyanionic cyclodextrin scaffold, has been evaluated for its utilization as a medical countermeasure (MCM) to neutralize the effects of fentanyl and related opioids. Initial *in vitro* toxicity assays demonstrate that SBX-Me has a nontoxic profile, comparable to the FDA-approved cyclodextrin-based drug Sugammadex. Pharmacokinetic analysis showed rapid clearance of SBX-Me with an elimination half-life of ∼7.4 h and little accumulation in major organs. SBX-Me was also evaluated for its ability to counteract the effects of fentanyl, carfentanil, and remifentanil in rats. Recovery times in rats exposed to sublethal fentanyl doses were found to be shorter when treated with SBX-Me after opioid exposure. The recovery times were reduced from ∼35 to ∼17 min for fentanyl, ∼172 to ∼59 min for carfentanil, and ∼18 to ∼12 min for remifentanil. SBX-Me increased the elimination half-life for fentanyl and remifentanil from 5.37 to 6.42 h and 8.24 to 9.74 h, respectively. These data support SBX-Me as a solid platform from which further research can be launched for the development of a MCM against the effects of fentanyl and its analogs. Furthermore, the data suggests that SBX-Me and other analogs are attractive candidates as broad spectrum opioids targeting MCMs.

## Abstract 2

Subetadex-α-methyl, a chemically modified polyanionic cyclodextrin, can bind and sequester synthetic opioids as a way to neutralize their toxic effects.

## Introduction

**Figure 1:**
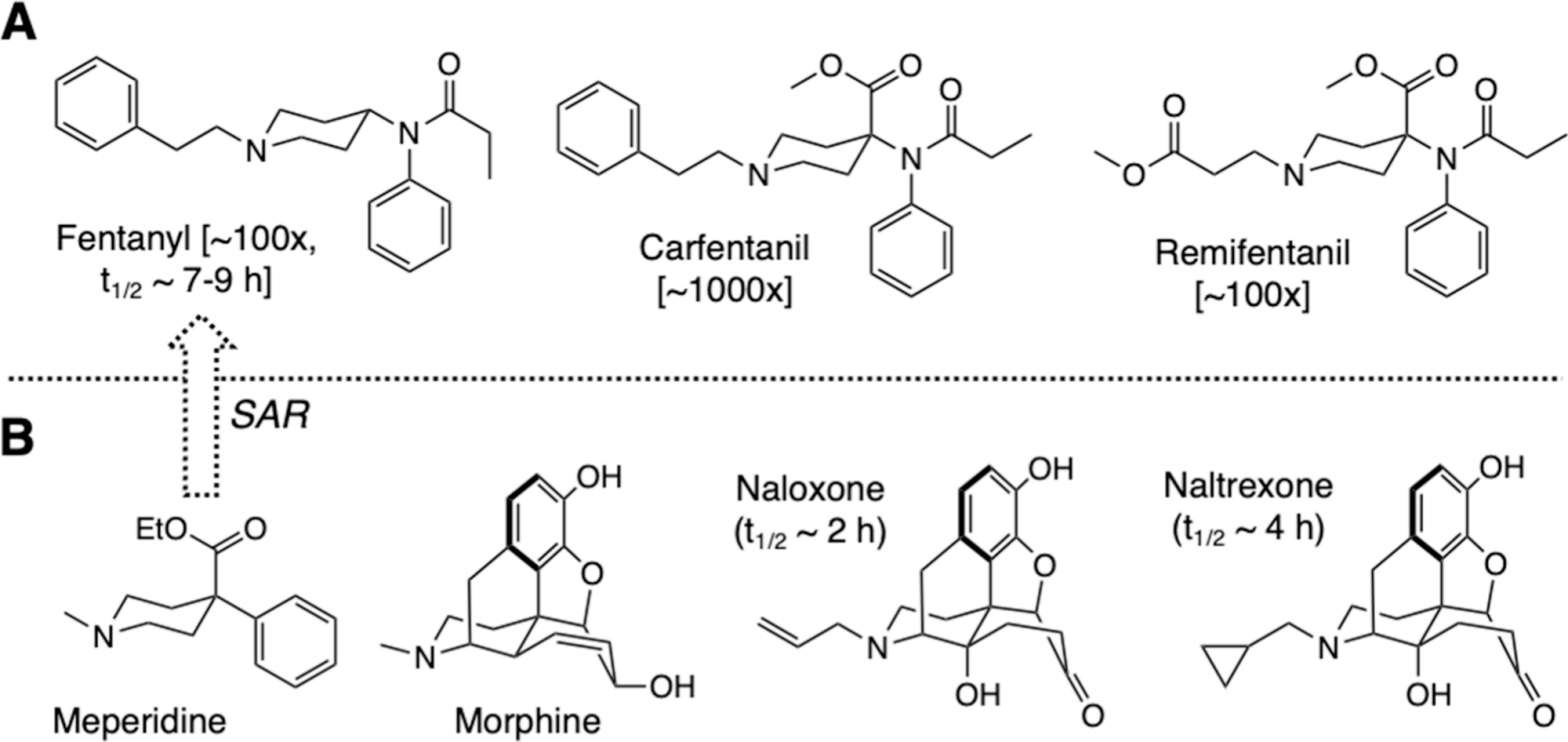
Structures of (A) fentanyl and its more and equally powerful analogs: carfentanil and remifentanil. Potencies of each relative to morphine are provided in brackets; (B) the original meperidine scaffold from which fentanyl originated via SAR studies along with the structure of morphine and the current state-of-the-art antidotes for overdose treatment: naloxone and naltrexone.

The global opioid pandemic has forced government agencies worldwide, as well as law enforcement entities, to strengthen their efforts in effectively combating the illicit manufacture and distribution of these lethal substances.^1,2^ One of the most common and often encountered synthetic opioid species is fentanyl, created in the laboratory of Paul Janssen over 60 years ago in an effort to discover more potent anesthetic compounds based on the meperidine scaffold^3,4^ (Figure 1). Since its discovery in 1960, fentanyl has been the subject of numerous structure–activity relationship studies that have yielded more powerful analogs, some of which have found beneficial clinical applications^5,6^ while others have become primary players in the world of illegal consumption.^7−10^ Another alarming part of the epidemic is the ease by which some of these analogs can be synthesized following protocols found openly on the Internet and in the scientific literature.^11−14^ Given the scale of this epidemic, drug and law enforcement agencies have established several ways of addressing the problem in order to mitigate its impact in society^15^ from developing more sensitive and rapid analytical detection techniques^16,17^ to the development of antidotes like naloxone and naltrexone.^18−20^ Another one deals with the development of medical countermeasures (MCMs) in the form of pretreatment methods that can provide an immediate layer of protection pre-exposure to the opioid.^21,22^

Two main characteristics for the development of a successful MCM include high specificity for the opioid and a large circulation half-life (preferably ∼5–6 days). Efforts toward attaining these goals have revolved around employing host molecules that could in principle capture synthetic opioids in their interior thereby neutralizing their effects on their biological target.^20^ One such class of host systems has been the cucurbit[n]urils, introduced by the Isaacs group at the University of Maryland for opioid-binding purposes, that has provided promising MCM candidates.^23,24^ Another set of similar hosts based on a cyclic carbohydrate backbone are the cyclodextrins (CDs), which unfortunately have been underexplored as MCM scaffolds for counter-fentanyl applications.^22,25^

**Figure 2:**
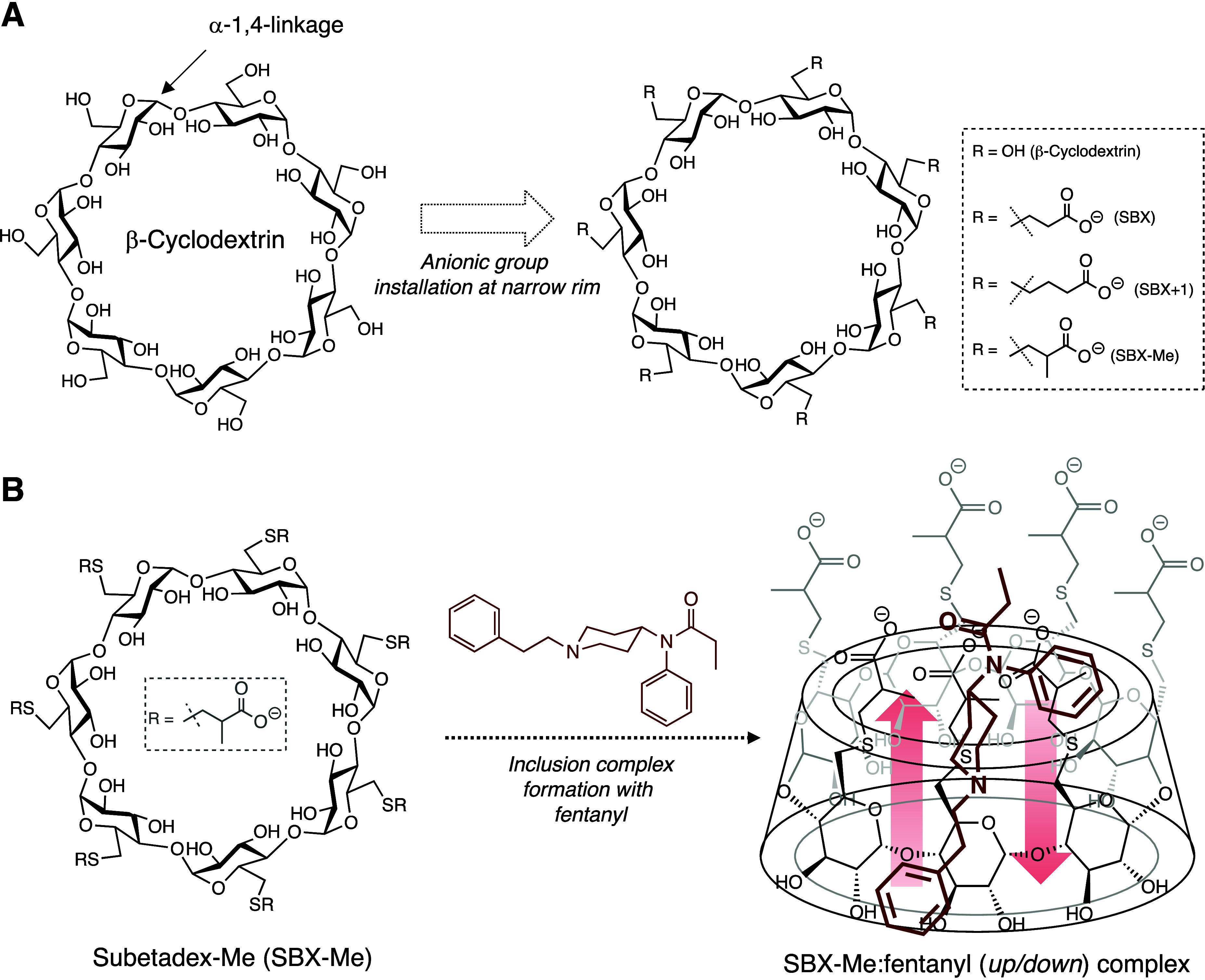
(A) Structure of β-cyclodextrin (β-CD) indicating the α-1,4-glycosidic linkages in a head to tail fashion between glucose units generate their cyclic nature along with three anionic β-CDs that have been evaluated as potential MCMs against fentanyl including SBX-Me. (B) Full structure of SBX-Me showing the anionic pendant units that serve to elongate and expand its hydrophobic interior and increase its hosting capability toward fentanyl and other synthetic opioids. Note that the opioid, fentanyl in this case, can bind in two orientations.

Cyclodextrins are cyclic oligosaccharide structures composed of glucose units joined together via α-1,4-glycosidic linkages (Figure 2a). These linkages give rise to a well-defined three-dimensional structure with a flexible cavity resembling that of a truncated cone open at both ends featuring a hydrophilic exterior and a much less hydrophilic interior.^26−28^ Historically, CDs have been attractive molecular scaffolds for their propensity to form inclusion complexes with organic molecules in host:guest interactions governed by mainly dispersion forces. In addition, CDs have raised interest in research and development ventures by serving as inexpensive, commercially available starting scaffolds that via established chemical modifications can be converted into new CDs with enhanced binding profiles. This type of host:guest association has found numerous applications in the pharmaceutical field, as the bioavailability, solubility, and stability of commonly used drugs have been enhanced because of their complexation with CDs.^29^ Thus, hydrophobic organic molecules of the right size will tend to form inclusion complexes with CDs at various binding strengths. For example, previous work in our lab resulted in the production of polyanionic β-CDs with enhanced binding affinity toward fentanyl relative to the native β-CD such as Subetadex (SBX) and Subetadex+1 (SBX+1) (Figure 2a).^30^ The work described herein summarizes the evaluation of another polyanionic β-CD based on SBX called Subetadex-α-methyl (SBX-Me), for its effectiveness in binding three synthetic opioids: fentanyl, carfentanil and remifentanil. These three synthetic opioids were chosen based on their toxicity profile, ease of manufacture, and propensity for illicit use. Although remifentanil is a fast-acting drug that quickly gets metabolized and may not pose the same safety concerns as fentanyl and carfentanil, it is still a powerful opioid with serious toxic side effects if the dose is not controlled. At higher doses acute overdose can be manifested by respiratory arrest. The polyanionic nature of SBX-Me originates from its pendant 2-methyl-3-thiopropanoic acid chains that line up its upper rim and due to their electrostatic interactions remain separated from each other (Figure 2b). As a result of this modification and newly given physical characteristic, the inner cavity of SBX-Me is expanded and can not only accommodate larger guests in its interior but also provide additional dispersion interactions that increase the strength of the host:guest inclusion complex with, for example fentanyl and analogs with longer features such as butyrylfentanyl or valerylfentanyl (Figure 2b). Relative to SBX, SBX-Me is a CD that possesses a chiral center on the thioalkylcarboxylate chains by the presence of the α-methyl group. As the starting thioalkylcarboxylic acid methyl ester used in the synthesis was racemic in nature, SBX-Me is a mixture of several isomers bearing the same molecular weight but providing very similar binding to the synthetic opioids used in this work.

## Results and Discussion

Development of effective MCMs against synthetic opioids like those belonging to the fentanyl class of compounds has become the focus of intense research by academic and industrial groups. The realization that a global pandemic involving these deadly substances is not only on the rise^31^ but without any potentially powerful solutions for its control has prompted the discovery and evaluation of not only effective treatments but also means by which a protective layer can be provided to an individual prior to an exposure to these compounds.^32^ Desirable characteristics for a MCM must include high specificity for the target toxic substance, blood circulation times in the order of 5–6 days and low systemic toxicity. Other more minor characteristics but not necessarily detrimental for eliminating a MCM from consideration include ease and inexpensive production cost for eventual mass production. To this end, an approach that has received heavy attention for this purpose is the use of molecular hosts that can encapsulate the fentanyl before it reaches its biological target. In this area, CDs have become a natural platform for this application based on their rich history as host molecules for a myriad of small molecules ranging from pharmaceutical drugs,^33−35^ toxic chemicals^36−38^ and biological molecules.^39^ In addition, the low toxicity associated with CDs and chemically modified analogs have increased their applications in the clinic, with Sugammadex (SGX) becoming the prime example of an FDA-approved CD drug for the reversal of anesthesia in patients suffering from its prolonged effects hours after a surgery.^40−44^

**Figure 3:**
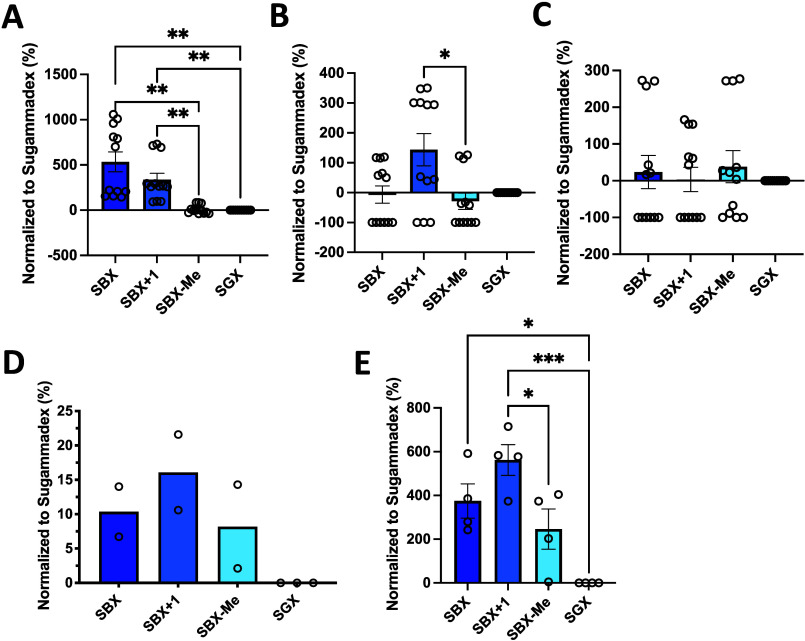
*In vitro* assessment of SBX and related CDs. (A–C) Hemolytic activity at 2 h; SBX, SBX+1, SBX-Me, and SGX at 10 (A), 1 (B) and 0.1 (C) mg/mL concentrations. Data is expressed as a percentage relative to the positive control (complete hemolysis). (D) Cholesterol solubilization data (*n* = 2/CD). (E) Release of phospholipids from hemoglobin-free human erythrocyte ghosts treated with the CDs. CD concentration was 25 mg/mL, and 2% Triton X-100 was used as a positive control. For all assays, data is normalized to SGX and is presented as the mean (±) of the standard error of the mean (SEM). A one-way ANOVA with Tukey post-test was run on all data with statistical significance noted for comparisons where *p < 0.05, **p < 0.01, and ***p < 0.001.

To this end, we embarked on a program to evaluate polyanionic CD analogs of SGX to find a candidate that could bind fentanyl and related analogs more tightly. These studies led to the discovery of polyanionic CD scaffolds with powerful binding constants for fentanyl such as Subetadex (SBX, K_a_ = 49,000 M^–1^). For polyanionic CD analogs that demonstrated favorable binding constants (K_a_ = 49,000 M^–1^ – K_a_ = 66,500 M^–1^^30^) initial *in* vitro assessments were conducted to evaluate potential toxicity; these assays were based on reported literature that have demonstrated that CDs can sequester cholesterol and induce hemolysis *in vitro* for various cells. These assays included: hemolytic activity, cholesterol and phospholipid solubilization. For our initial assessments, we benchmarked the outcomes for our CD analogs to FDA approved and clinically used SGX, given its FDA approval and use in the clinic. The results from these initial *in vitro* assessments of SBX and related analogs^30^ are shown in Figure 3. Hemolytic activity was assessed in human erythrocytes for 10, 1, and 0.1 mg/mL of SBX/analog/SGX and is shown in Figure 3, panels A-C (10, 1, and 0.1 mg/mL), respectively. At the highest concentration (10 mg/mL), significant differences were noted between SBX (534.2% ± 110.1), SBX+1 (338.1% ± 68.9) and SBX-Me (7.7% ± 13.8), as well as when comparing both SBX and SBX+1 to SGX (p-values shown in Figure 3) when normalized to SGX. For lower concentrations (1 and 0.1 mg/mL), no statistical significance was noted between any groups except SBX+1 vs SBX-Me at 1 mg/mL (*p* < 0.05). Cholesterol solubilization is shown in Figure 3D, where when normalized to SGX, SBX-Me demonstrated the lowest solubilization (8.2% ± 6.1) compared to both SBX (10.4% ± 3.6) and SBX+1 (16.1% ± 5.5). Finally, in panel E, phospholipid solubilization is shown. Statistical differences were noted when comparing SBX (374.7% ± 78.4) and SBX+1 (562.1% ± 70.4) to SGX; no significant difference was observed when comparing SBX-Me to SGX. These initial *in vitro* results together with NMR results clearly showing a strong binding between SBX-Me and fentanyl (See SI for discussion and experimental data) demonstrated that the most favorable CD analog compared to SGX was SBX-Me. Considering the strong binding affinity for SBX-Me and fentanyl together with the similar nontoxic profile compared to SGX, SBX-Me was advanced for further *in vivo* evaluation. As a side note it is important to mention that SBX-Me bears a chiral center in its pendant thioalkylcarboxylate chains. As the synthesis of SBX-Me involved the use of a racemic mixture of the mercaptoalkylcarboxylic acid methyl ester, the SBX-Me used in this work is actually a mixture of several isomers. However, we do not anticipate that the nature of the stereocenter in the alkyl chains will have a profound effect on the overall binding of the CD to the opioids. Nevertheless, enantiomerically pure versions of the methyl esters of the mercaptoalkylcarboxylic acids are commercially available and may be used to provide enantiomerically pure SBX-Me.

### Pharmacokinetics of SBX-Me

**Figure 4:**
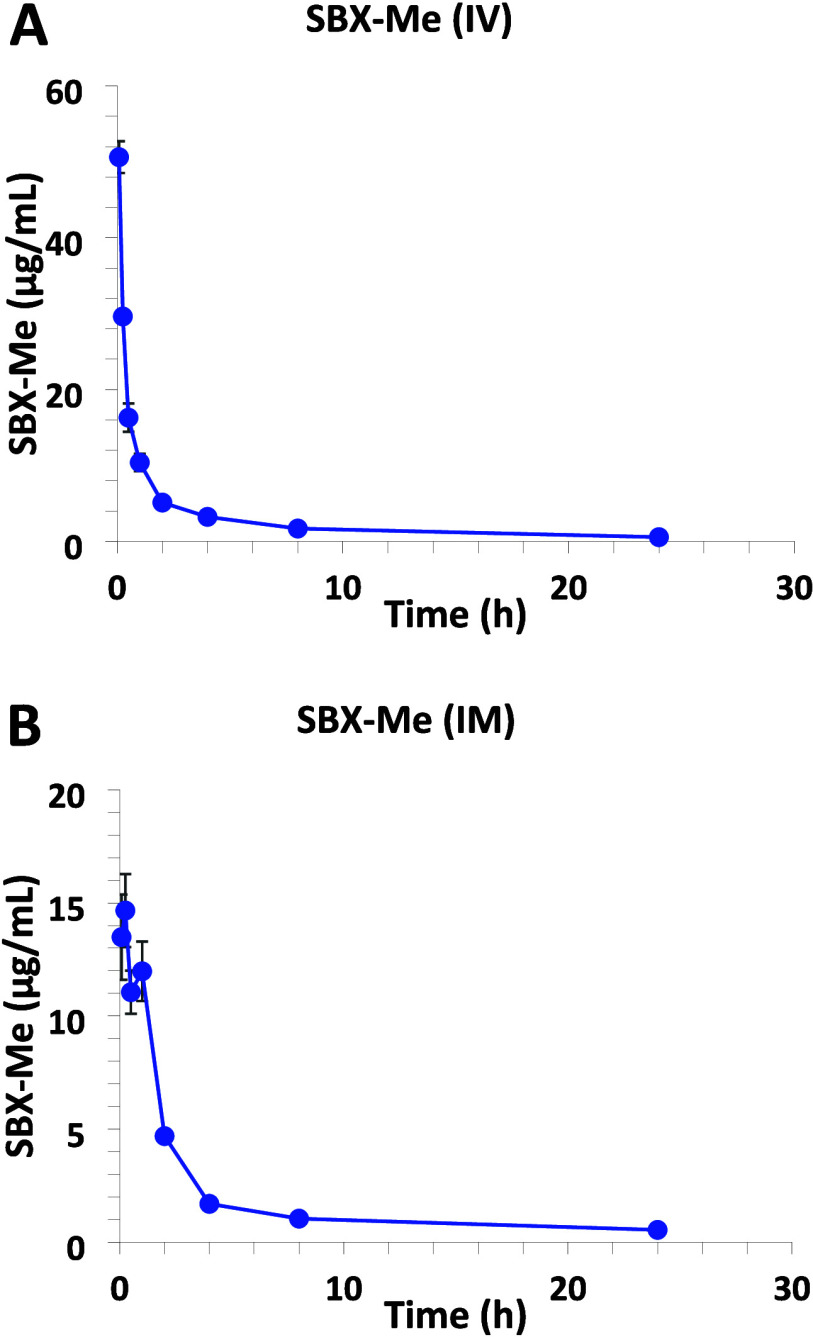
Mean plasma concentration–time profiles of SBX-Me following (A) single intravenous (IV) or (B) intramuscular (IM) administration of 16 mg/kg ^14^C-SBX-Me to male Sprague–Dawley rats. Data are expressed as the mean of 5 animals ± the standard error.

Pharmacokinetic parameters of ^14^C-SBX-Me were evaluated for both IV and IM dose routes. Mean plasma concentrations over time of SBX-Me are illustrated in Figure 4 and the mean PK parameters are presented in Table 1. The plasma concentration versus time curve for both dose routes were similar following first order kinetics. A comparison of PK parameters from IV administration versus IM administration of a 16 mg/kg dose of SBX-Me showed a significant lower maximum observed concentration (*C*_max_), following the IM exposure (Table 1). Intramuscular exposed animals had a longer distribution half-life (*t*_1/2dist_) of 0.83 h. compared to the IV exposure of 0.33 h. The elimination half-life (*t*_1/2elim_) was twice as long in the IM exposed animals when compared to the IV exposures (Table 1). The observed *t*_max_ from the IM exposure was 0.3 h indicating rapid absorption from the injection site into the plasma compartment (data not shown). Total clearance of SBX-Me from plasma (CL) was 220.5 and 306.4 mL/h/kg and the apparent volume of distribution (V_d_) was 2349.2 and 5896.3 mL/kg for the IV and IM doses respectively, suggesting rapid and extensive distribution beyond the plasma compartment. The lower CL and V_d_ in the IV exposed animals indicate a higher proportion of the compound remained in the plasma of the IV exposed animals. The mean AUC_0-t_ was 66.9 and 42.0 ± 2.41 μg•h/mL for the IV and IM dose route, respectively (Table 1). Based on the difference in AUC_0-t_ between the two dose routes the absolute bioavailability (F) was determined to be 0.63, indicating abundant absorption into the systemic circulation after an IM exposure.

**Table 1:** Mean Pharmacokinetic Parameters of SBX-Me Following a Single Administration of 16 mg/kg ^14^C-SBX-Me to Male Sprague Dawley Rats^a^

dose (mg/kg)	*C*_max_ (μg/mL)	*t*_1/2dist_ (h)	*t*_1/2elim_ (h)	AUC_0-t_ (μg·h/mL)	AUC_0-inf_ (μg·h/mL)	*V*_d_ (mL/kg)	CL (mL/h/kg)
16, IV	49.8 ± 4.4	0.33 ± 0.32	7.38 ± 0.46	66.9 ± 4.76	72.7 ± 4.54	2349.2 ± 231	220.5 ± 13.9
16, IM	14.7 ± 2.6	0.83 ± 0.18	14.71 ± 2.6	42.0 ± 7.71	56.5 ± 20.5	5896.3 ± 523	306.4 ± 102

a Data are expressed as the mean of 5 animals ± the standard deviation. IV: intravenous, IM: intramuscular.

### Pharmacokinetics of SBX-Me with Opioid Challenge

**Figure 5:**
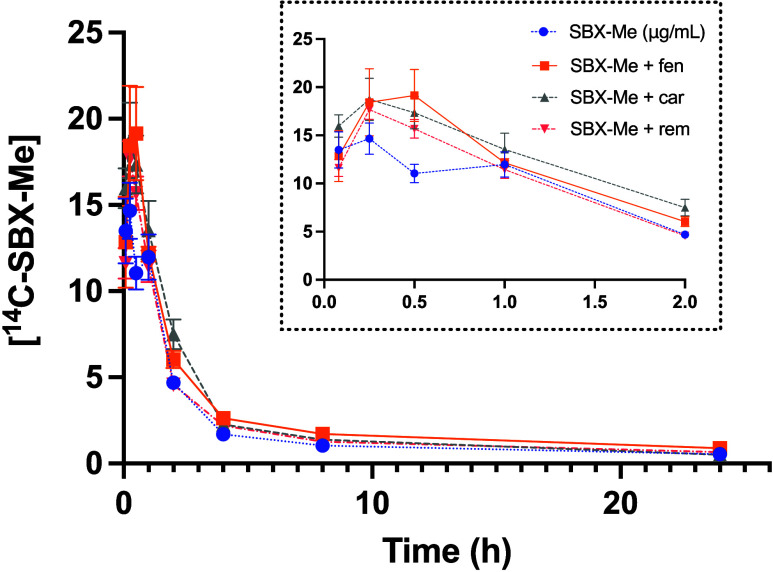
Mean plasma concentration–time profiles of an IM exposure of ^14^C-SBX-Me (16 mg/kg) after an IV exposure to fentanyl (50 μg/kg), carfentanil (5 μg/kg), or remifentanil (5 μg/kg) in male Sprague–Dawley rats. Inset is a zoom in of earlier time points (0–2 h). Data are expressed as the mean of *n* = 5 animals ± the standard error.

To determine if opioids affect the kinetics of SBX-Me, the pharmacokinetic parameters of SBX-Me were evaluated after an IV exposure of either fentanyl, carfentanil or remifentanil followed by treatment with an IM dose of ^14^C-SBX-Me in Sprague–Dawley rats. Mean plasma concentrations over time of SBX-Me are illustrated in Figure 5 and the mean PK parameters are presented in Table 2. The plasma concentration versus time curve for SBX-Me with and without fentanyl, carfentanil or remifentanil were similar following first order kinetics. Results show that there was no significant change in SBX-Me plasma concentration or PK parameters indicating that the three opioids that were evaluated do not significantly interfere with SBX-Me pharmacokinetics or clearance.

**Table 2:** Mean Pharmacokinetic Profiles of an IM Exposure of ^14^C-SBX-Me after an IV Exposure to Fentanyl, Carfentanil, or Remifentanil in Male Sprague Dawley Rats^a^

dose^b^	*C*_max_ (μg/mL)	*t*_1/2dist_ (h)	*t*_1/2elim_ (h)	*t*_max_ (h)	AUC_0-t_ (μg·h/mL)	AUC_0-inf_ (μg·h/mL)	*V*_d_ (mL/kg)	CL (mL/h/kg)
SBX-Me	14.7 ± 2.6	0.83 ± 0.18	14.71 ± 2.6	0.3 ± 0.0	42.0 ± 7.71	56.5 ± 20.50	5896.3 ± 523	306.4 ± 102
SBX-Me + fentanyl	18.2 ± 5.90	0.70 ± 0.24	14.1 ± 2.72	0.45 ± 0.1	63.3 ± 13.80	82.2 ± 23.30	4071.3 ± 553.6	207.7 ± 63.4
SBX-Me + carfentanil	17.5 ± 3.17	1.27 ± 0.62	10.2 ± 2.04	0.40 ± 0.11	54.8 ± 8.42	62.8 ± 13.23	3783.8 ± 270.5	263.5 ± 56.6
SBX-Me + remifentanil	18.7 ± 2.11	0.57 ± 0.06	10.1 ± 1.06	0.40 ± 0.11	46.9 ± 2.60	53.7 ± 3.76	4448.7 ± 453.2	263.3 ± 80.8

a Data are expressed as the mean of 5 animals ± the standard deviation.

b ^14^C-SBX-Me dose = 16 mg/kg (IM); Fentanyl dose = 50 μg/kg (IV); Carfentanil dose = 5 μg/kg (IV); Remifentanil dose = 5 μg/kg (IV).

### Tissue Distribution of SBX-Me

When adjusted to total tissue weight, at the *C*_max_, the liver contained the highest proportion of the dose with a mean of 5.2% of the administered dose followed by the kidney at 2.5%. All other tissues contained less than 1% of the administered dose (See Supporting Information). No SBX-Me was detected in the brain. In the heart and lung SBX-Me was rapidly eliminated from the tissue whereas in the liver, kidney, and spleen elimination was significantly slower over the course of the experiment. As expected, the levels of SBX-Me in the tissues from the IM exposed animals were lower compared to the IV dosed animals (See Supporting Information).^45,46^ There was no significant difference in tissue distribution or concentration of SBX-Me in the presence of the opioids when compared to the results obtained from exposure to SBX-Me alone (See Supporting Information). The relatively higher levels and slower elimination rate of SBX-Me in the kidneys could increase the potential for toxic effects since previous studies have shown that certain cyclodextrins have been shown to induce nephrotoxic properties. Some α- and β-CDs have been shown to be nephrotoxic by manifesting a series of alterations in the vacuolar organelles of the proximal tubule after parenteral administration.^47^ Additionally, Sugammadex caused a histopathological degeneration in the kidneys when administered to rats at therapeutic dose levels. However, these changes did not cause any deterioration in renal function and were deemed to not be a safety concern.^48^

### Pharmacokinetics of Opioids

**Figure 6:**
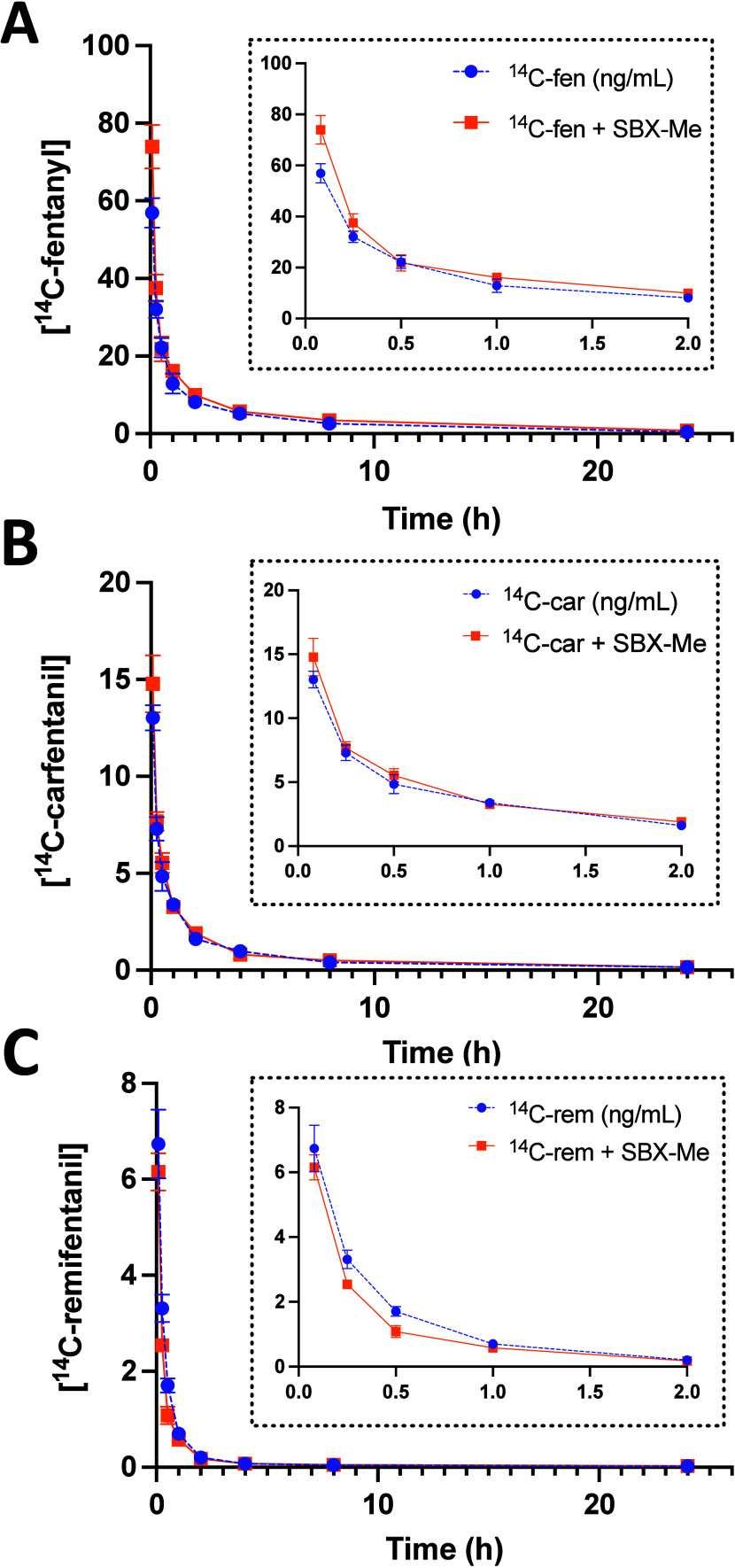
Mean plasma concentration–time profiles of (A) fentanyl, (B) carfentanil, and (C) remifentanil following single intravenous (IV) administrations of ^14^C-fentanyl (50 mg/kg), ^14^C-carfentanil (5 mg/kg), or ^14^C-remifentanil (50 mg/kg) and (IM) administrations of 16 mg/kg SBX-Me to male Sprague–Dawley rats. Insets are a zoom in of earlier time points (*t* = 0–2 h.). Data are expressed as the mean of *n* = 5 animals ± the standard error.

To determine if SBX-Me affects the pharmacokinetics of the studied opioids, the pharmacokinetic parameters of fentanyl, carfentanil, and remifentanil were evaluated after exposure to ^14^C-fentanyl, ^14^C-carfentanil, or ^14^C-remifentanil and treatment with SBX-Me. Mean plasma concentrations over time of fentanyl, carfentanil, and remifentanil are illustrated in Figure 6 and the mean PK parameters are presented in Table 3. The plasma concentration versus time curve for fentanyl, carfentanil, and remifentanil were similar following first order kinetics. Both the distribution and elimination half-lives were similar for both fentanyl and carfentanil treatment groups even thought there was a 10-fold difference in dose between fentanyl and carfentanil. The elimination half-life for remifentanil was about 35% longer than the other two opioids. The elimination rate constants (CL/V_d_) for fentanyl and carfentanil were similar at 0.107 and 0.109, respectively indicating similar rates of movement throughout the body. The elimination rate constant for remifentanil was smaller at 0.071. The observed increase in the elimination half-lives of 6.42 ± 0.38 h and 9.74 ± 0.96 h for fentanyl and remifentanil with SBX-Me treatment respectively, were statistically different when compared to the elimination half-lives for fentanyl (5.37 ± 0.52 h) and remifentanil (8.24 ± 0.69 h) exposure without SBX-Me treatment, indicating that the presence of SBX-Me may alter the elimination half-lives of these opioids (Table 4). It is presumed that the lack of significance in the elimination half-lives from carfentanil was due to the relatively large variation between the individual animals from these treatments resulting in an increase in the standard deviation between the treatment groups.

**Table 3:** Mean Pharmacokinetic Parameters of Fentanyl, Carfentanil, and Remifentanil with and without SBX-Me Treatment in Male Sprague Dawley Rats^a^

treatment group^b^	*C*_initial_ (μg/mL)	*t*_1/2dist_ (h)	*t*_1/2elim_ (h)	AUC_0-t_ (ng·h/mL)	AUC_0-inf_ (ng·h/mL)	*V*_d_ (mL/kg)	CL (mL/h/kg)
Fentanyl	51.6 ± 7.63	0.35 ± 0.04	5.37 ± 0.52	89.85 ± 9.09	92.8 ± 8.36	3893.5 ± 911	502.1 ± 99.2
Fentanyl + SBX-Me	64.4 ± 13.52	0.30 ± 0.03	6.42 ± 0.38^c^	116.64 ± 7.31	124.18 ± 6.95	3749.62 ± 399	403.6 ± 23.1
Carfentanil	11.76 ± 1.61	0.40 ± 0.09	7.93 ± 2.06	18.72 ± 2.58	20.66 ± 2.11	2834 ± 926	255.5 ± 25.4
Carfentanil + SBX-Me	13.6 ± 3.65	0.35 ± 0.05	6.28 ± 2.11	18.42 ± 1.67	20.35 ± 1.70	2249.45 ± 819	246.8 ± 20.2
Remifentanil	6.88 ± 1.63	0.27 ± 0.02	8.24 ± 0.69	4.08 ± 0.69	4.34 ± 0.72	14118 ± 2998	1186 ± 244.3
Remifentanil + SBX-Me	5.36 ± 0.67	0.26 ± 0.02	9.74 ± 0.96^c^	3.56 ± 0.27	3.94 ± 0.39	17908 ± 699	1282 ± 120.8

a Data are expressed as the mean of 5 animals ± the standard deviation.

b ^14^C-Fentanyl dose = 50 mg/kg (IV),^14^C-carfentanil dose = 5 mg/kg (IV),^14^C-remifentanil dose = 5 mg/kg (IV), and SBX-Me dose = 16 mg/kg (IM).

c Statistically different from the opioid exposure group without SBX-Me treatment. *p* < 0.05.

**Table 4:** Recovery Times in Male Rats after Exposure to Fentanyl, Carfentanil, and Remifentanil with and without Treatment with SBX-Me

treatment^a^	time to recovery
fentanyl	35 min
fentanyl + SBX-Me	17 min
carfentanil	172 min
carfentanil + SBX-Me	59 min
remifentanil	18 min
remifentanil + SBX-Me	12 min

a Fentanyl dose = 50 mg/kg (IV), carfentanil dose = 5 mg/kg (IV), remifentanil dose= 5 mg/kg (IV), and SBX-Me dose = 16 mg/kg (IM).

### Tissue Distribution of Fentanyl and Its Derivative

Both fentanyl and carfentanil were detected in all tissues examined (See Supporting Information). Opioid levels rapidly declined from each tissue over the time course of the experiment with background levels returning at approximately 24 h. SBX-Me did not appear to have any effect on the tissue disposition of the opioids.

### Metabolism of SBX-Me/Fentanyls

HPLC analysis of urine from rats exposed to 16 mg/kg ^14^C-SBX-Me revealed that SBX-Me was excreted unchanged over the 24-h period at a retention of 3–4 min which corresponded to the retention time of an SBX-Me standard. Exposure to fentanyl, carfentanil, or remifentanil did not alter the SBX-Me urinary profiles of the animals. Analysis of the urine of rats exposed to 50 μg/kg ^14^C-fentanyl, 5 μg/kg ^14^C-carfentanil or 5 μg/kg ^14^C-remifentanil followed 1 min later with SBX-Me revealed changes in the metabolic profiles of the opioids suggesting the formation of an opioid/SBX-Me complex. Urinary radioprofiles showed a shift in the HPLC retention time of ^14^C-fentanyl from 8 min to 3–4 min (See Supporting Information). Likewise, a similar shift for carfentanil and remifentanil was observed (from 13 min to 3–4 min and from 14 to 4 min, respectively). The radioactive peaks detected at 3–4 min corresponded to the retention time of ^14^C-SBX-Me suggesting that the opioids formed a complex with SBX-Me *in vivo* and were excreted in the urine with a retention time similar to SBX-Me (Supporting Information).

### Effect of SBX-me on Fentanyl Intoxication Recovery Time

In addition to the PK/ADME studies, SBX-Me was evaluated *in vivo* for its ability to counteract the effects of fentanyl, carfentanil and remifentanil in rats. Animals were observed after opioid exposure for any physiological changes and the time to recovery from opioid intoxication with and without SBX-Me treatment. Recovery was determined when animals could right themselves and freely move about their cages. For all three opioids tested, treatment with SBX-Me decreased the time it took animals to recover compared to animals that did not receive the CD (Table 4). The observed recovery times were reduced from ∼35 to ∼17 min for fentanyl, from ∼172 to ∼59 min for carfentanil and from ∼18 to ∼12 min for remifentanil. These results indicate that SBX-Me can decrease the effects of opioid intoxication by aiding in the recovery after sublethal exposures to the three opioids tested.

### Discussion

A new, polyanionic, cyclodextrin scaffold known as Subetadex-α-methyl (SBX-Me) has been identified and subsequently evaluated for use as a potentially useful medical countermeasure to combat the effects of fentanyl and related opioids. Preliminary *in vitro* toxicity assays including hemolytic activity and cholesterol binding propensity for SBX-Me demonstrated its nontoxic profile relative to other promising CD analogs promoting it into the next phase involving *in vivo* PK and biodistribution studies. Initially, PK studies, employing a ^14^C-radiolabeled version of SBX-Me, showed a rapid clearance of SBX-Me with an elimination half-life of *t*_1/2_ ∼ 7.4 h with little accumulation in major organs (e.g., heart, liver, spleen, kidney and lung). SBX-Me was not detected in the brain. Metabolism and clearance analysis showed that SBX-Me was excreted in the urine unchanged. Subsequent *in vivo* experiments demonstrated the ability of SBX-Me to alter the PK parameters and metabolic profiles of fentanyl, carfentanil, and remifentanil when administered after opioid exposure in Sprague–Dawley rats. Treatment with SBX-Me caused an increase in the elimination half-lives of the opioids, matching them closely to the elimination half-life of the SBX-Me itself strongly suggesting the formation of a SBX-Me:fentanyl complex. This concept was further reinforced with the observation from the HPLC urinary metabolic profiles showing altered opioid retention times matching that of SBX-Me after SBX-Me treatment. By altering the PK profile (i.e., increasing the half-life) the rate and extent of absorption of the opioid can be altered which can alter the dose that reaches the target tissues potentially leading to decreased toxicity. Furthermore, by forming an SBX-Me/opioid complex the change in metabolism and clearance can influence the concentration of the opioid in the body and the duration of its exposure to various organs and tissues which could ultimately alter the toxicity profile of the compound. The PK and metabolism result together with the decrease in recovery times from animals treated with SBX-Me suggest that this cyclodextrin may influence the toxicity profile of these synthetic opioids by sequestering the free opioid, preventing the drug from interacting with the target receptors therefore neutralizing its effects, which would serve to lower the effective lethal dose of fentanyls leading to a reduced risk from exposure. This proposed mechanism is different compared to conventional treatments where a competitive antagonist (i.e naloxone) competes with the opioids for binding sites on the receptors^49^ but is similar to that of the related cyclodextrin Sugammadex in which the neuromuscular blocking agent rocuronium is deactivated by encapsulation by the cyclodextrin.^50^ In contrast, the present finding that fentanyl and carfentanil was detected in brain tissue when dosed together with SBX-Me dissuades the argument that an SBX-Me/opioid complex is formed since data shows that SBX-Me alone was not detected in brain tissue. To speculate, SBX-Me may limit the amount of drug getting to the brain but not totally eliminate brain penetration by sequestering some of the free opioid before it crosses the blood–brain barrier. More studies are needed to fully elucidate the toxicity profile of SBX-Me, and interactions between SBX-Me and this class of opioids, and their effects on SBX-Me/opioid biodisposition and brain penetration. These additional studies should include determining the crystal structure of the fentanyl-SBX-Me inclusion complex to better understand the host–guest interactions.^51^ These studies can provide direct insight into the binding mechanism of the fentanyl molecule and cyclodextrin and the functional mechanism of the inclusion complex which can influence stability, solubility and drug efficacy. Previous studies have determined the crystal structure of an inclusion complex of β-cyclodextrin and fentanyl, and together with molecular confirmational calculations showed that fentanyl is totally contained within the β-cyclodextrin cavity and that the phenylethyl part of fentanyl existed in several conformations.^51^ Overall, the results presented herein collectively present SBX-Me as a plausible starting platform for further development as a MCM in the treatment of fentanyl poisoning. Due to its ability to sequester not only fentanyl but other synthetic opioids such as carfentanil and remifentanil and confer some degree of protection in animals, it is hoped that after additional studies SBX-Me can be further advanced as a potential broad spectrum MCM against a variety of synthetic opioids.

## Materials and Methods

### Chemicals and Reagents

Reagents and solvents were purchased and used as received. Acetone, methanol, *N*-methyl-2-pyrrolidinone (NMP), sodium hydroxide and hydrogen sulfide were purchased from VWR (Radnor, PA.). Heptakis-6-deoxy-6-bromo-β-cyclodextrin was purchased from Arachem/Cyclodextrin Shop (Tilberg, Netherlands). 2-Methyl methacrylate was purchased from BeanTown Chemicals (Hudson, NH.). 1-Hydroxybenzotriazole (HOBT), diisopropylcarbodiimide (DIC) and anhydrous dimethylformamide were purchased from Sigma-Aldrich (St. Louis, MO.). ^14^C-Radiolabeled versions of 2-methyl methacrylic acid and propionic acid were purchased from American Radiolabeled Chemicals, Inc., (St. Louis, MO). Thiol-mediated displacement reactions on the C6-per-brominated β-cyclodextrin followed by methyl ester hydrolysis was accomplished with a modified version of the originally published protocol.^40,52^ For the synthesis of the cold and ^14^C-radiolabeled mercaptoalkylcarboxylic acid methyl ester intermediates, thin layer chromatography (TLC) was used to monitor their production using Merck kieselgel 60-F_254_ glass sheets and detection accomplished with development of color using ceric ammonium molybdate (CAM) and iodine vapor.^53,54^ The three ^14^C-radiolabeled fentanyls (fentanyl, carfentanil, remifentanil) were synthesized as previously reported^13^ but using the final acylation step to introduce the ^14^C-radiolabel onto the opioids. Multimilligram (∼200–300 mgs) synthesis of SBX-Me was accomplished in similar fashion as for the synthesis of SBX,^30^ and then purchased in multigram quantities (2 × 10G) from Cyclolab Kft. (Budapest, Hungary). The purification of all intermediates, regular as well as ^14^C-radiolabeled, was accomplished by flash column chromatography using a Biotage Isolera purification system using Biotage Sfär silica high-capacity duo cartridges (10 G).

### Synthesis of ^14^C-Fentanyl, ^14^C-Carfentanil, and ^14^C-Remifentanil

**Scheme 1:**
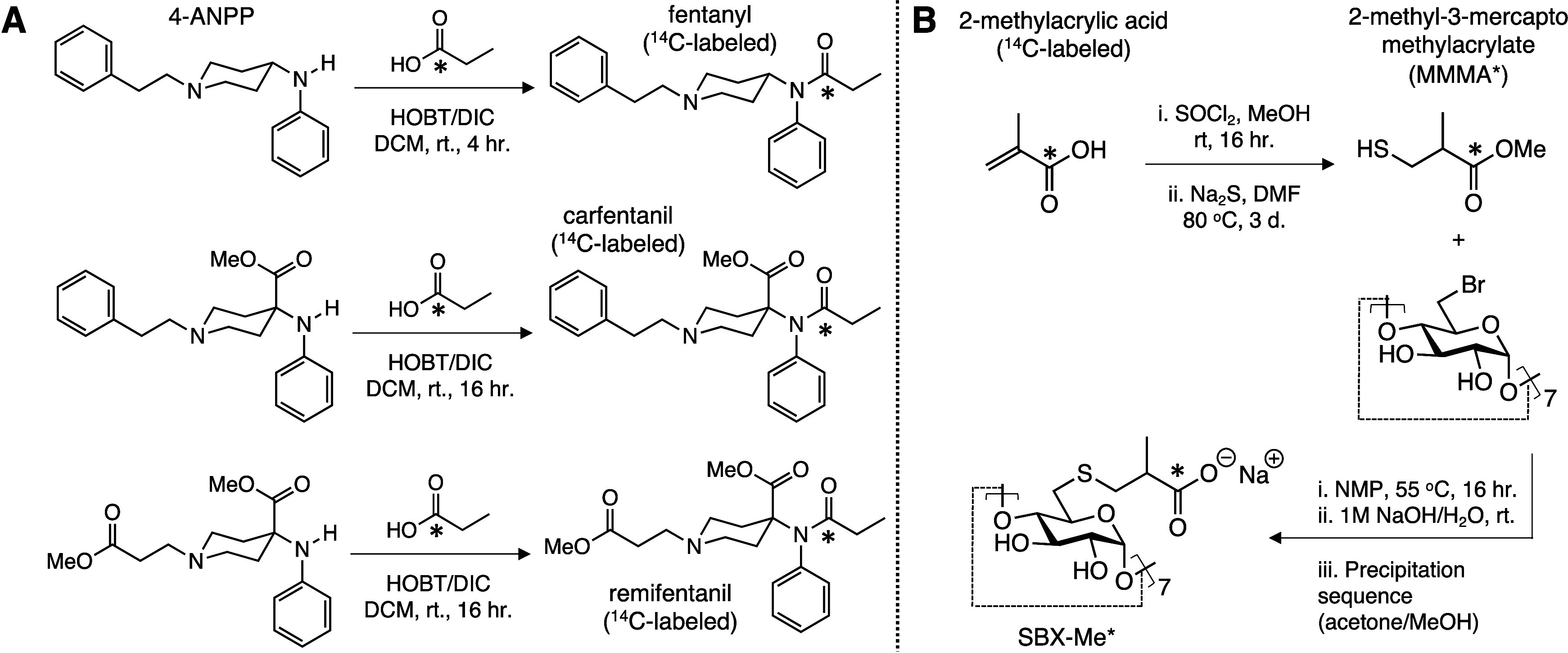
Synthesis of (a) ^14^C-Labeled Fentanyl, Carfentanil, and Remifentanil and (b) ^14^C-Labeled SBX-Me (SBX-Me*) The asterisk (*) shows the carbon atom where the ^14^C-label is in each molecule.

The syntheses involve acylation as the last step in order to minimize handling of the valuable ^14^C-radiolabeled propionic acid, and also to minimize the loss of any radiolabeling efficiency in the final product during the early steps due to either low reaction yields or competing side reactions. Thus, reaction between 4-ANPP and ^14^C-labeled propionic acid (radiolabel at the carbonyl atom) using HOBT/DIC (1-hydroxybenzotriazole and diisopropylcarbodiimide) as coupling reagents provides the ^14^C-radiolabeled fentanyl. For the remaining two opioids, carfentanil and remifentanil, the ^14^C-radiolabel was introduced analogously at the acylation step using the precursors on Scheme 1a. The only differences lie in the amount of time for the reaction to take place. All the ^14^C-radiolabeled opioids were purified using flash column silica gel chromatography (DCM → 3:7 MeOH/DCM).

### Synthesis of ^14^C-SBX-Me (SBX-Me*)

As there are no commercial vendors for ^14^C-radiolabeled 2-methyl methacrylate, a synthetic route was devised for its production from the commercially available ^14^C-radiolabeled 2-methyl methacrylic acid. The synthesis of ^14^C-radiolabeled 3-mercapto-2-methyl methylpropanoate is outlined in Scheme 1b, and it involves the conversion of ^14^C-radiolabeled 2-methyl methacrylic acid to its acyl chloride using thionyl chloride and esterification with methanol. With the ^14^C-radiolabeled 2-methyl methacrylate, several conditions were tested for the Michael addition using the mercapto anion (See Supporting Information). The most optimal conditions for the synthesis of ^14^C-radiolabeled 3-mercapto-2-methyl methylpropanoate involved the heating (80 °C) of the methyl methacrylate in wet DMF in the presence of Na_2_S to furnish the desired ^14^C-radiolabeled side chain in 77% yield after purification. Reaction of ^14^C-radiolabeled 3-mercapto-2-methyl methylpropanoate with the C6-deoxy-6-heptabromo-β-CD in NMP at 55 °C overnight to provide the ^14^C-radiolabeled SBX-Me (SBX-Me*) as an off-white flaky solid after two alternating rounds of precipitation from MeOH, water, and acetone respectively. The methyl ester hydrolysis was accomplished using a 1 M NaOH/H_2_O solution and stirring overnight at ambient temperature followed by precipitation using acetone to yield white flakes that were collected by centrifugation (Scheme 1b). Additionally, the white solid was resuspended, washed with MeOH (2 × 50 mL) and collected by centrifugation. Lastly, the solid was vacuum filtered, washed with MeOH (2 × 20 mL), and dried under vacuum for 2 h. This method of purification is the same that we have used in the synthesis of SBX and previously described SBX analogs.^30^

### EI-GC-MS Analysis Method

A 6890 Agilent GC with 5975 MS detector equipped with a split/splitless injector was used for analysis of all intermediate small molecules. The GC column used for the analysis was an Agilent HP-5 ms UI capillary column (30 m × 0.25 mm id ×0.25 μm film thickness). Ultrahigh purity helium, at 0.8 mL/min, served as the carrier gas. The inlet was operated in pulsed splitless mode (25 psi for 1 min, followed by a 50 mL/min purge flow), with the injector temperature set at 250 °C and the injection volume was 1 μL. The oven temperature program was as follows: 40 °C, held for 3 min, increased at 8 °C/min to 300 °C, held for 3 min. The MS ion source and quadrupole temperatures were 230 and 150 °C, respectively. Electron ionization (EI) was used with an ionization energy of 70 eV. The MS was operated to scan from *m*/*z* = 29 to 600 in 0.4 s with a solvent delay of 3.5 min as previously described.^55,56^

### *In Vitro* Assays

Assays included hemolytic activity, cholesterol solubilization and solubilization of phospholipids. The concentration range of the CDs in the in vitro assays were based on reported literature values from previous studies with known CD analogs.^57−59^ A one-way ANOVA with Tukey post-test was run on all data with statistical significance noted for comparisons where *p* < 0.05.

### Hemolytic Activity

Hemolytic activity was assessed using established protocols.^57^ Erythrocytes were separated from citrated human blood (500*g*, 5 min, 3X PBS wash) and resuspended in PBS. The CDs, SBX, SBX-Me, Sugammadex (SGX), and SBX+1 (where the side chains possess and additional methylene unit relative to SBX) were added to the cell suspension (16% v/v in PBS), gently mixed and incubated at 37 °C. At 2 h, samples were centrifuged at 500 x *g* and supernatants were collected. The absorbance of hemoglobin was measured in the supernatant using λ = 577 nm with λ = 655 nm as a reference wavelength. The percent of hemolysis is expressed as the ratio of hemoglobin in each sample supernatant relative to hemoglobin concentration after complete hemolysis of cells in Triton x-100.

### Cholesterol Solubilization

Cholesterol solubilization was assessed using established protocols.^58^ The CDs (SBX, SBX-Me, SGX, and SBX+1) were dissolved in PBS (10 mM) and an excess of cholesterol (100 mM) was added. Suspensions were mixed at room temperature for 12 h. After incubation, suspensions were filtered (Co-star, SpinX, 0.22 μm filters) and the cholesterol contents of filtrates were determined by ultrahigh performance liquid chromatography (UPLC) using the following method: isocratic, flow rate of 0.250 mL/min, mobile phase: acetonitrile/isopropanol (50/50), column: Phenomenex C18 Synergi Max RP 250 × 4.6, column temperature of 55 °C, U*V*_max_ of λ = 207 nm. Percent solubilization is calculated based on the peak areas from the CD/cholesterol solution evaluated relative to the cholesterol standard solution and all data (n = 2/CD) are normalized to SGX.

### Solubilization of Phospholipids

Phospholipid solubilization was assessed using established protocols.^59^ To assess the solubilization of phospholipids for CDs, hemoglobin-free human erythrocyte ghosts were used since hemoglobin interferes with the colorimetric assay for phospholipoids. Ghost cell suspensions (0.250 mL) were added to a Tris-HCl (10 mM) buffer containing the CDs (SBX-Me, SBX, SGX, and SBX+1). The samples were incubated in a 37 °C water bath for 1 h. and then centrifuged at 2000*g* for 20 min at 4 °C. The amount of phospholipid in the supernatant was determined using the commercially available Phospholipid Assay Kit (MAK122, Sigma-Aldrich, St Louis, MO, λ = 570 nm).

### *In Vivo* Assessment

Animal experiments were conducted at the Lawrence Livermore National Laboratory (LLNL) AAALAC accredited animal care facility. The protocol for the animal experiments was reviewed and approved by the LLNL Institutional Animal Care and Use Committee (IACUC) prior to the initiation of the study. All experiments were performed in accordance with relevant guidelines set forth by the Guide for the Care and Use of Laboratory Animals (Eighth Ed.). In addition, the study was carried out in compliance with the ARRIVE guidelines. Male Sprague–Dawley rats weighing 250–300 g were obtained from Envigo Laboratories (Indianapolis, IN.). Rats were housed individually in polystyrene cages containing hardwood bedding and kept on a 12-h. light/dark cycle in a ventilated room maintained at 24 °C. Food and water were provided *ad libitum*.

### Pharmacokinetics of SBX-Me

To evaluate the pharmacokinetics (PK) of SBX-Me, male Sprague–Dawley rats were administered a single intravenous (IV) or intramuscular (IM) dose of ^14^C-SBX-Me dissolved in sterile saline at a concentration of 16 mg/kg, which is the therapeutic dose for the FDA-approved cyclodextrin SGX. At the designated time points of 0.08, 0.25, 0.5, 1, 2, 4, 8, and 24 h. postexposure, whole blood was collected, and the plasma was separated within 1 h of collection by centrifugation. The volume of plasma obtained was recorded and the sample was stored at −80 °C until analysis. Total radiocarbon content of the plasma samples was quantified by accelerator mass spectrometry (AMS) as described previously.^60^

The plasma PK parameters of SBX-Me were calculated by noncompartmental analysis using PK Solutions software (Summit Research Services, Montrose, CO.). A two-stage approach was used to independently fit the plasma concentration data from each rat, and then determine the individual means ± standard errors. The half-life (*t*_1/2_) and the initial concentration observed in plasma (C_0_) were determined from the concentration-versus-time data. The area under the concentration vs time curve (AUC) was calculated for the intervals from time zero to time t (AUC_0-t_), where t is the time of the last measurable concentration (24 h.), and for time zero to infinity (AUC_0-inf_), using the linear trapezoidal method. The volume of distribution (V_d_) was determined on the basis of the AUC determination and reflects the V_d_ during the elimination phase. The clearance (CL) calculation is based on the AUC0_0-inf_. These parameters provided valuable information on bioavailability, the effect of dose route on clearance, and the effect of dose on the extent of absorption beyond the central compartment. To assess the effect these three fentanyls have on SBX-Me pharmacokinetics, the above procedure was repeated except that one minute prior to the ^14^C-SBX-Me dose animals received an IV dose of either fentanyl (50 μg/kg), carfentanil (5 μg/kg), or remifentanil (5 μg/kg) through an implanted jugular vein catheter. The dose concentrations of the opioids were chosen to provide a nonlethal maximum observed anesthetic effect.^61−63^

### Biodistribution of SBX-Me

Biodistribution of SBX-Me was determined as described previously.^63^ Briefly, rats (n = 5 per time point) were administered a single IM dose of 16 mg/kg ^14^C-SBX-Me with or without an IV dose of fentanyl (50 μg/kg), carfentanil (5 μg/kg), or remifentanil (5 μg/kg), as described above. Following dose administration, animals were euthanized by CO_2_ asphyxiation at 0.08, 0.25, 0.5, 1, 2, 4, 8, and 24 h. post dose. Immediately following euthanasia, whole animal perfusion (heparinized (50 K U/L) PBS), was performed to ensure all the blood was removed from the tissues prior to collection. After perfusion, tissues (brain, liver, kidney, heart, spleen and lung) were excised from the carcass and rinsed twice in PBS. All tissues were then stored in glass vials (28 × 60 mm) at −80 °C until analysis for carbon-14 content by AMS.^64^ To assess metabolism of ^14^C-SBX-Me a subset of animals was placed in metabolism cages and urine was collected for 24 h. after ^14^C-SBX-Me administration. The collected urine was analyzed by reversed-phase HPLC to determine the presence of SBX-Me metabolites or breakdown products.

### Biodisposition of Fentanyl, Carfentanil, and Remifentanil

To assess the affect SBX-Me has on opioid biodisposition animals were exposed to an IV dose of either ^14^C-fentanyl (50 μg/kg), ^14^C-carfentanil (5 μg/kg), or ^14^C-remifentanil (5 μg/kg) through an implanted jugular vein catheter followed one minute later with an IM dose of SBX-Me (16 mg/kg). At the designated time points of 0.08, 0.25, 0.5, 1, 2, 4, 8, and 24 h. postexposure, blood and urine was collected, for PK and metabolism analysis of each opioid, as described above.

### Safety Statement

The synthesis of synthetic opioids should be executed by specially trained personnel and always involve the use of the appropriate personal protective equipment (PPE) that includes lab coat, nitrile gloves underneath butyl gloves, face shield, and eye protection. All the synthetic steps leading to the formation of the final opioid should take place in a well-ventilated chemical fume hood for maximum shielding from the chemicals. In addition, Narcan antidote should always be present nearby for rapid use when these synthetic and analytical standard preparations are taking place.

## Supporting Information Available

Complete synthetic methods and characterization of SBX-Me, ^14^C-radiolabeled SBX-Me, fentanyl, carfentanil, and remifentanil; NMR spectra of SBX-Me; experimental methods and additional data including tissue distribution profiles and metabolism studies (PDF)

## Dedication

This article is dedicated to the memory of our colleague Victoria Hio Leong Lao.
