# Supplementary material for: Evaluation of Subetadex-α-methyl, a Polyanionic Cyclodextrin Scaffold, as a Medical Countermeasure against Fentanyl and Related Opioids
Source: ACS Cent Sci. 2024 Oct 23;10(12):2200–12. doi: 10.1021/acscentsci.4c00682 (PMC11672541; doi:10.1021/acscentsci.4c00682)
Supplement: Supplementary file 1 — oc4c00682_si_001.pdf [file oc4c00682_si_001.pdf]

Evaluation of Subetadex-α-methyl (SBX-Me), a polyanionic cyclodextrin scaffold, as a medical countermeasure against fentanyl and related opioids

Michael A. Malfatti[1,2]*, Heather A. Enright[1,2], Summer McCloy[1,2], Esther A. Ubick[1,2], Edward Kuhn[1,2], Alagu Subramanian[1-4], Victoria Hio Leong Lao[1,2], Doris Lam[1,2], Nicholas A. Be[1,2], Saphon Hok[3,4], Edmund Y. Lau,[1,2] Derrick C. Kaseman,[1,4,5] Brian P. Mayer,[1,3,4] Carlos A. Valdez[1,2,4]*

[1]Physical and Life Sciences Directorate, [2]Biosciences and Biotechnology Division, [3]Global Security Directorate, [4]Forensic Science Center, [5]Materials Science Division, Lawrence Livermore National Laboratory, Livermore, CA, 94550, USA.

-Supporting Information-

Table of Contents

Synthesis of SBX-Me	S2
Synthesis of ^{14}C-radiolabeled fentanyls and SBX-Me	S2
Synthesis of 3-mercapto-2-methyl methylpropanoate	S3
Synthesis of ^{14}C-SBX-Me	S4
^{1}H-NMR spectra of SBX-Me	S6
^{13}C-NMR spectra of SBX-Me	S7
^{13}C-DEPT-135-NMR spectra of SBX-Me	S8
ROESY NMR Characterization	S9
Figure S1. 1D NMR spectra of fentanyl, SBX-Me, and fentanyl + SBX-Me	S10
Figure S2. 1D ROESY spectra with irradiation of the fentanyl aromatic protons and the SBX-Me methyl signature	S11
Figure S3. 2D ROESY of the fentanyl+SBX-Me and fentanyl	S12
Figure S4. Tissue distribution profile for SBX-Me	S13
Figure S5. Tissue distribution profile for SBX-Me with added fentanyl (IV, 50 mg/kg) and carfentanil (IV, 5 mg/kg)	S14
Figure S6. Tissue distribution profile for fentanyl and carfentanil with added SBX-Me (IV, 16 mg/kg)	S15
Metabolism studies on SBX-Me treated animals	S16
Figure S7. Urinary metabolic radioprofiles of fentanyl, carfentanil and remifentanil	S17
References	S18

Synthesis of SBX-Me

Heptakis-6-bromo-6-deoxy-β-cyclodextrin (1.0 g, 0.63 mmol) was made into a suspension in *N*-methyl-2-pyrrolidone (NMP, 20 mL) in a 100 mL round bottomed flask equipped with a stir bar. To this solution, cesium carbonate (2.06 g, 6.3 mmol, 10 equiv. to cyclodextrin) was added in small portions followed by the addition via syringe of methyl 3-mercapto-2-methylpropanoate (0.56 mL, 0.67 g, 6.3 mmol, 10 equiv. to cyclodextrin). The resulting mixture was heated to 55 ^{o}C overnight with vigorous stirring. The following day, the suspension was cooled to ambient temperature and the mixture added dropwise to a vigorously stirring 500 mL Erlenmeyer flask filled with 300 mL of acetone to induce precipitation of the modified cyclodextrin. Stirring of the suspension was done at ambient temperature for 10 minutes and the white precipitate was collected by centrifugation. The white solid was then washed with deionized water (2 x 50 mL) and each time collected by centrifugation. The precipitate was taken up in acetone (50 mL) and vacuum filtered through a fritted disc filter (medium porosity) and dried under vacuum for 30 minutes to furnish the methyl ester β-cyclodextrin intermediate as a pure, off-white solid (924 mg, 84%). The methyl ester β-cyclodextrin intermediate (924 mg, 0.52 mmol) was treated with 1 M NaOH/H_2O (3.90 mL, 3.90 mmol, 7.5 equiv. to cyclodextrin) in a 20 mL scintillation vial equipped with a stir bar. The initial suspension became a full solution (light tan in color) after 10 minutes of stirring at ambient temperature. The mixture was vigorously stirred overnight. The light tan solution was added dropwise to a stirring acetone bath (300 mL) in a 500 mL Erlenmeyer flask. White flakes precipitated out upon the dropwise addition of the mixture and these were collected by centrifugation. Additionally, the white solid was re-suspended, washed with MeOH (2 x 50 mL) and collected by centrifugation. Lastly, the solid was vacuum filtered, washed with MeOH (2 x 20 mL) and dried under vacuum for 2 h. The procedure yielded pure SBX-Me (896 mg, 86%). ^{1}H NMR (D_2O, 600 MHz) δ 5.20-5.14 (br m, 7H), 3.99-3.90 (br m, 14H), 3.63-3.56 (br m, 7H), 3.09-3.06 (br m, 7H), 2.96-2.92 (br m, 7H), 2.87-2.84 (br m, 7H), 2.62-2.58 (br m, 7H), 2.52-2.48 (br m, 7H), 1.16-1.13 (br m, 21H); ^{13}C NMR (D_2O, 150 MHz) δ 183.9 (C=O, 99.8 (br), 82.4 (br), 72.9, 72.0 (br), 71.1 (br), 43.3, 37.0 (CH_2), 34.0 (CH_2), 17.5 (CH_3); LC-MS(TOF): for $C_{70}H_{110}O_{42}S_7$ $[M-2H]^{2-}$, *m/z* = 923.2269 (923.2264 calc.).

Synthesis of ^{14}C-radiolabeled fentanyls and SBX-Me

Synthesis of ^{14}C-fentanyl, ^{14}C-carfentanil and ^{14}C-remifentanil - For ^{14}C-fentanyl*, the precursor 4-ANPP was reacted with a ^{14}C-labeled propionic acid (radiolabel at the carbonyl atom) that was activated using HOBT/DIC (hydroxybenzotriazole and diisopropylcarbodiimide) to yield the ^{14}C-labeled fentanyl (fentanyl*) (Scheme S1a). The fentanyl* was purified using flash column silica gel chromatography using a Biotage Purification System. The syntheses of carfentanil* and remifentanil* were accomplished in similar fashion in one step starting from the amine precursor and using the same coupling protocol used for fentanyl* (Schemes S1b and S1c).

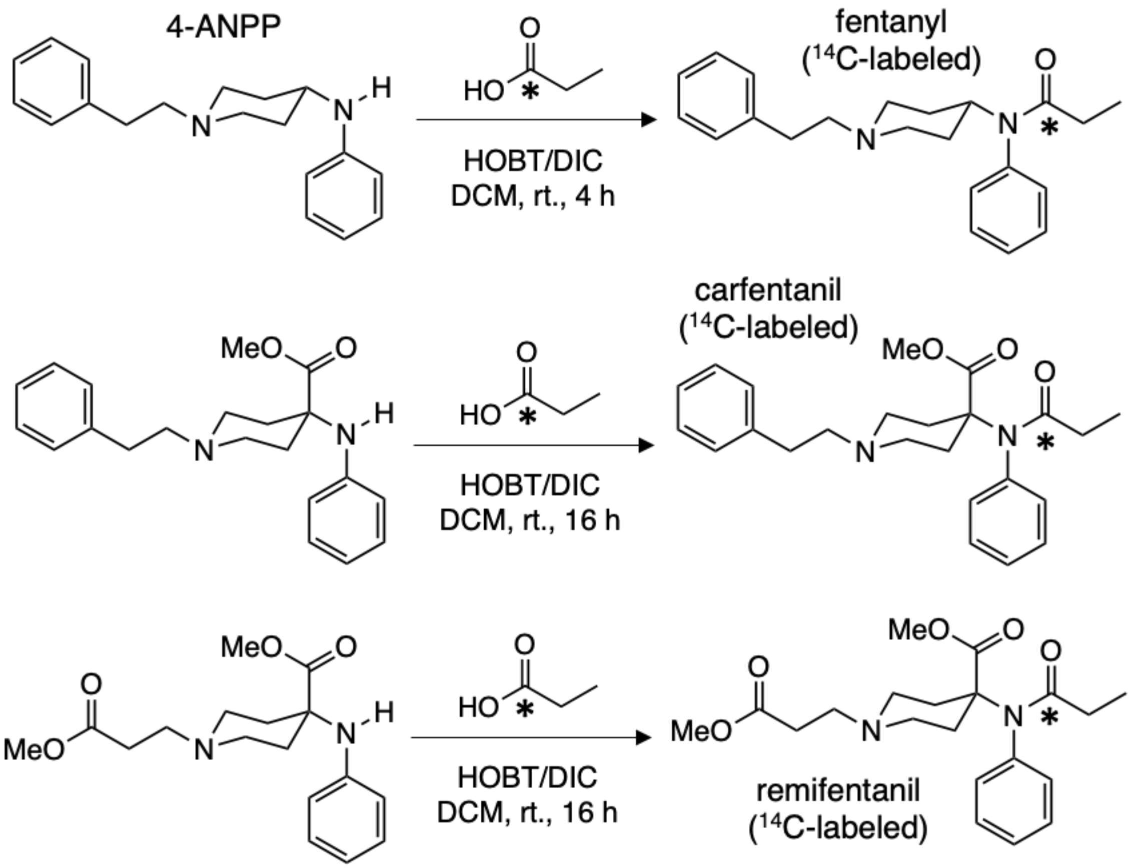

Scheme S1. (a) Synthesis of ^{14}C-radiolabeled fentanyl, (b) carfentanil and (c) remifentanil. The location of the radiolabeling in each opioid is indicated by the asterisk.

Synthesis of ^{14}C-3-mercapto-2-methyl methylpropanoate – Often the synthesis of radiolabeled materials is done using well established, high yielding transformations in addition to the already discussed important caveat of introducing the radiolabel preferably in the last step of a synthetic route. In our situation, we worked on optimizing a route to 3-mercapto-2-methyl methylpropanoate and subsequently used these conditions when the more expensive, radiolabeled version of 3-mercapto-2-methyl methylpropanoate was to be synthesized.

The preparation of 3-mercapto-2-methyl methylpropanoate is outlined in Scheme S2a. For the synthesis of this material, the unavailability of the intermediate 2-methyl methacrylate (Scheme S2a, dashed box) in its radiolabeled version forced us to explore its synthesis from 2-methyl acrylic acid. Thus, esterification of 2-methyl acrylic acid using thionyl chloride and methanol furnished 2-methyl methacrylate in 96% yield after column purification. Conversion of 2-methyl methacrylate to the final product 3-mercapto-2-methyl methylpropanoate was accomplished via its heating with sodium thiosulfate in DMF. The 3-mercapto-2-methyl methylpropanoate was obtained in 72% after column purification (Scheme S2a). After working out the most optimal conditions for the synthesis of 'cold' 3-mercapto-2-methyl methylpropanoate, we turned our attention to the synthesis of its 'hot' or ^{14}C-radiolabeled version that is outlined in Scheme S2b below. A solution of methyl methacrylate (0.1 g, 0.1 mmol) and ^{14}C-methyl methacrylate (1mCi, 1mCi/vial, 10mCi/mL methanol, 58 mCi/mmol, cat. ARC-4250-1mCi) was mixed with H_2S (0.8

M in THF, 2.0 mL, 1.5 mmol, 1.5 equiv.) and NH_3-MeOH (methanolic ammonia - 2M solution, 0.8 mL) in MeOH (3 mL) in a 40 mL RB flask equipped with a stir bar. The flask was equipped with a condenser and was refluxed at 100 °C overnight. The following day, the flask was cooled to ambient temperature and its content was partitioned in DCM (10mL) and H_2O (10 mL) and separated. The aqueous phase was washed with DMC (5mL x 2) and combined to the organic phase. The organic phase was washed with brine (10 mL), dried over anhydrous $MgSO_4$, filtered, and evaporated to yield a light brown residue that was purified by flash column chromatography (Biotage, hexanes → 10% EtOAc/hexanes → 20% EtOAc/hexanes) to give ^{14}C-labelled 3-mercapto-2-methyl-methylpropanoate (150mg). The purified product was confirmed by GC/MS. The yields obtained for the radiolabeled version of 3-mercapto-2-methyl methylpropanoate was obtained in 70% yield over two steps and purification using flash column chromatography.

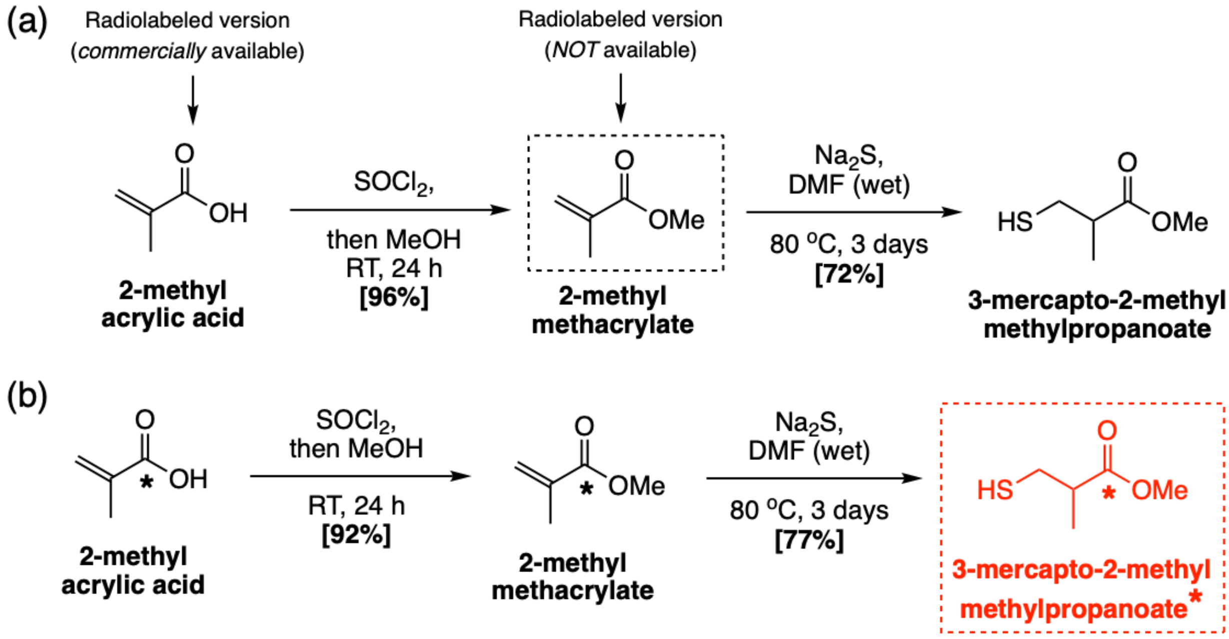

Scheme S2. (a) Syntheses of 'cold' 3-mercapto-2-methylpropanoate and (b) its 'hot', ^{14}C-radiolabled version.

Synthesis of ^{14}C-SBX-Me - The final step in the synthesis of ^{14}C-SBX-Me (SBX-Me*) involves the reaction between the radiolabeled 3-mercapto-2-methylpropanoate* and a commercially available heptabrominated-b-CD as outlined in Scheme S3 below. The radiolabeled 3-mercapto-2-methylpropanoate* was diluted (1:10) with cold 3-mercapto-2-methylpropanoate in order to lessen the amount of radioactivity in the final product. In a capped 20-mL vial, a mixture of heptabrominated-b-CD (120mg), Na_2CO_3 (0.3g), 3-mercapto-2-methyl-methylpropanoate (360mg) and ^{14}C-labelled 3-mercapto-2-methyl-methylpropanoate (60mg) in DMF (3mL) was heated (65 °C) for 3 days. Afterward, the reaction mixture was cooled to ambient temperature and drop-by-drop added into vigorously stirred acetone (200mL). Next, the solid was collected by centrifugation and washed with water (1mL, 3x) and washed with acetone (1mL, 3x). Each washing steps involved centrifugation and decanting off the liquids. The collected solid (60mg) was then treated with 1 M NaOH (1mL) and stirred at room temperature overnight. Next day, the mixture was drop-by-drop added into methanol (20mL), stirred vigorously for 1-2 hours to precipitate out the target product, and allow to stand undisturbed overnight. Next, methanol was

decanted off, the solid was collected by centrifugation and repeatedly washed with acetone (1mL x 5). After vacuum drying, the sodium salt of ^{14}C-SBX-Me (15 mg) was collected with a radiopurity >95%. The reaction yields the material in decent yield (between 35-41%) after several rounds of purification of the SBX-Me* involving precipitation from acetone, water, methanol, then acetone again followed in each step by centrifugation. This method of purification is the same that we have used in the synthesis of cold SBX-Me and other SBX analogs as previously described[1, 2].

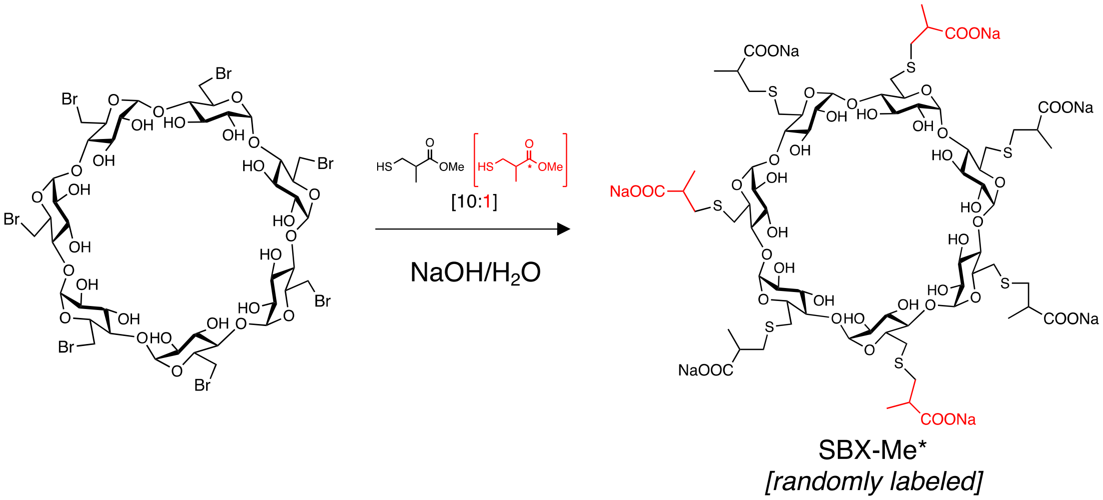

Scheme S3. Synthesis of SBX-Me* (Na$^+$ form). The red labeling of SBX-Me only indicates one of the many combinations that the radiolabeled molecule may feature in terms of substitution number and patterns.

^{1}H NMR for SBX-Me

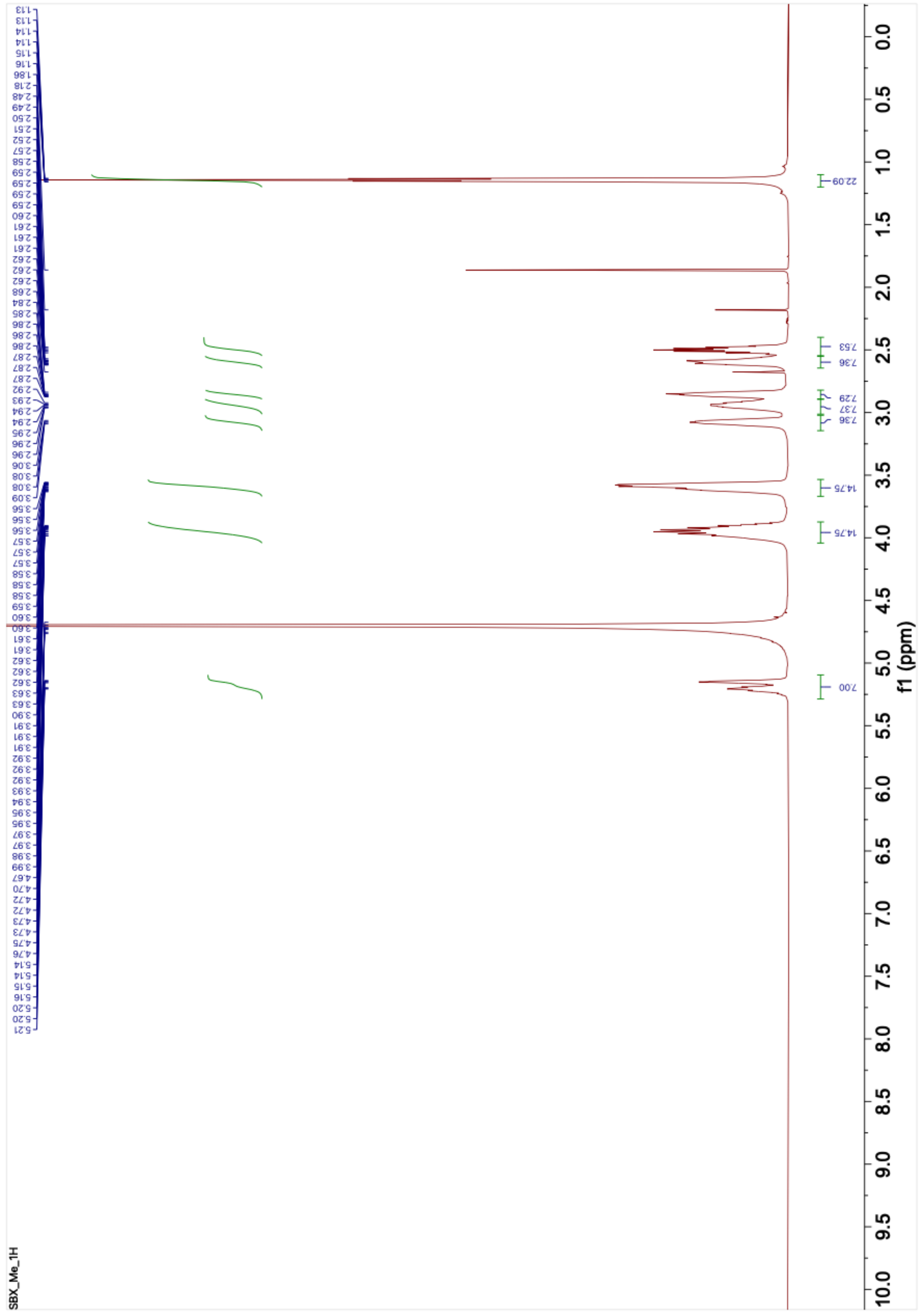

^{13}C-NMR for SBX-Me

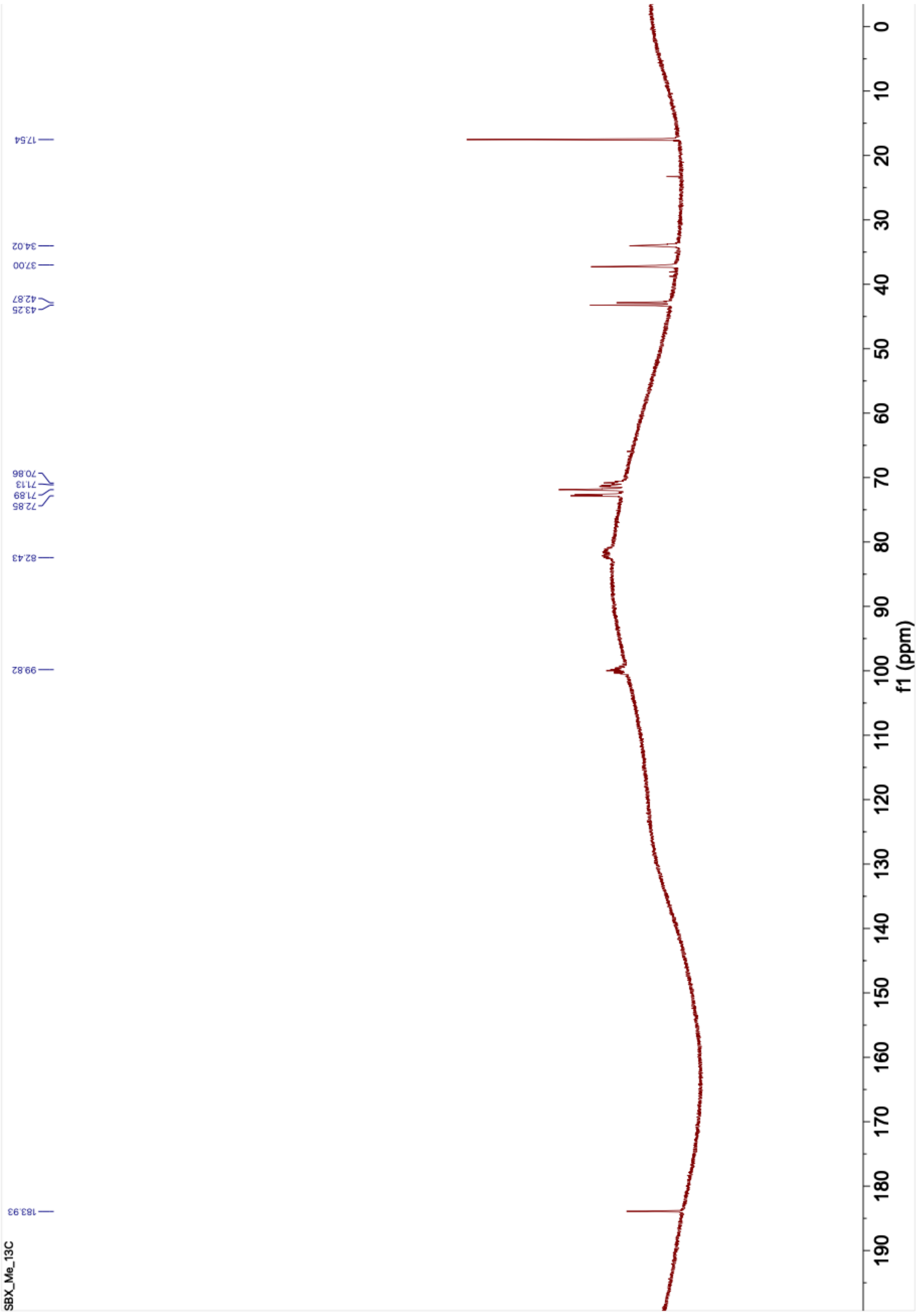

^{13}C-DEPT-135-NMR for SBX-Me

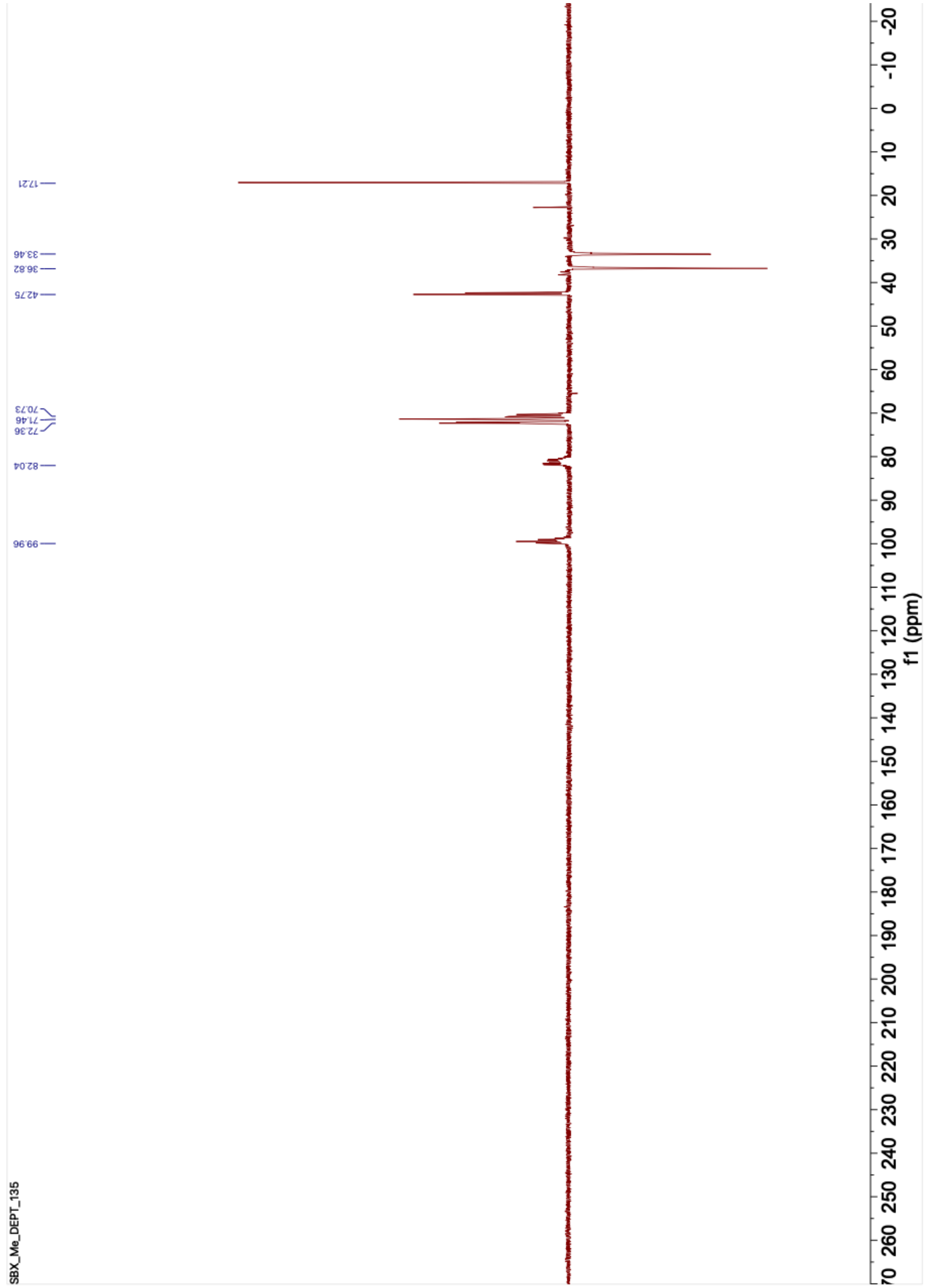

ROESY NMR Characterization

Experimental details

Experimental: NMR Spectroscopy was performed using a Bruker Avance III Spectrometer operating at 14.1 T (^{1}H=500.994 MHz). 1D Pulse acquire experiments was collected on the SBX-Me and fentanyl samples using a 30-degree tip angle (4 µs), a 3.72 s recycle delay, and 16 averages. Due to the low concentrations of analyte in the fentanyl+SBX-Me (10:1 ratio) were collected using a presaturation sequence to remove the residual protons in the D_2O solvent. In this sample, a 90-degree pulse was used with a 3.72 s recycle delay, and 16 averages. Rotating frame Overhauser Effect Spectroscopy (ROESY) NMR spectra were collected in selective 1D and 2D fashions. In both experiments a spin-lock of 2.5 ms was used. The 1D spectra selectively irradiated either the fentanyl aromatic proton signatures (~7.32 ppm) or the methyl signature of the SBX-Me (1.046 ppm) with 10,000-16,384 averages collected. 2D ROESY measurements were collected on SBX-Me, fentanyl, and fentanyl+SBX-Me with 238-436 t1 increments with 40-240 averages per t_1 increment.

Results and Discussion

The 1D spectra of the fentanyl, SBX-Me, and 10:1 fentanyl:SBX-Me are shown in Figure S5. Notable features include the aromatic protons of fentanyl near 7.4 ppm, and the resonances of the thiol group, which are noted on the figure. The other resonances in the SBX-Me spectra correspond to the interior of the SBX-Me cyclodextrin. To probe binding interactions, ROESY NMR was used, which is sensitive to distances <1 nm. 2 case studies were performed using 1D selective ROESY in which only a portion of the NMR spectrum is irradiated (negative peaks) and any ROESY interactions appear as positive peaks. Figure S6 shows the 1D selective ROESY spectra irradiated at 7.32 ppm (aromatic protons) and 1.046 (methyl signature on SBX-Me). The appearance of positive peaks near upon irradiation of the aromatic fentanyl protons shows ROESY interactions with both the cyclodextrin core and the thiol. Irradiating the methyl signature of the thiol shows the appearance of the aromatic protons. Together these results indicate that fentanyl is bound to the SBX-Me, but these results do not indicate a preferential arrangement of the fentanyl in the cyclodextrin. 2D ROESY (Figure S7) was performed to investigate the bonding. Multiple cross peaks are present and the peaks at 1.49, 1.96, and 2.87 are intermolecular between different chemical shifts within the fentanyl. The aromatic region of the fentanyl shows new correlations to chemical shifts at 3.71 and 1.04 ppm, which correspond to the cyclodextrin core and the methyl group of the thiol, further supporting the 1D selective ROESY measurements and indicative of binding between the two species.

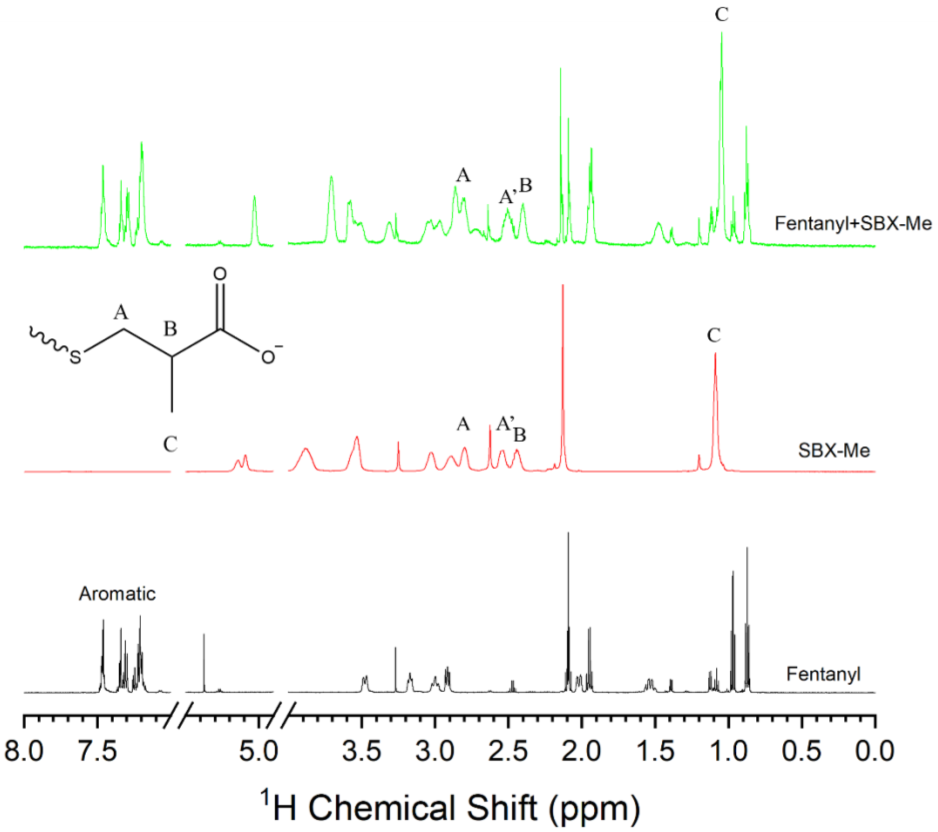

Figure S1. 1D NMR spectra of fentanyl (bottom), SBX-Me (middle), and a 10:1 ratio of fentanyl and SBX-Me (top). The resonances of the thiol group SBX-Me are denoted by A,B, and C or the structure and the NMR spectra. The designation of A and A' distinguish between the prochiral CH_2 protons.

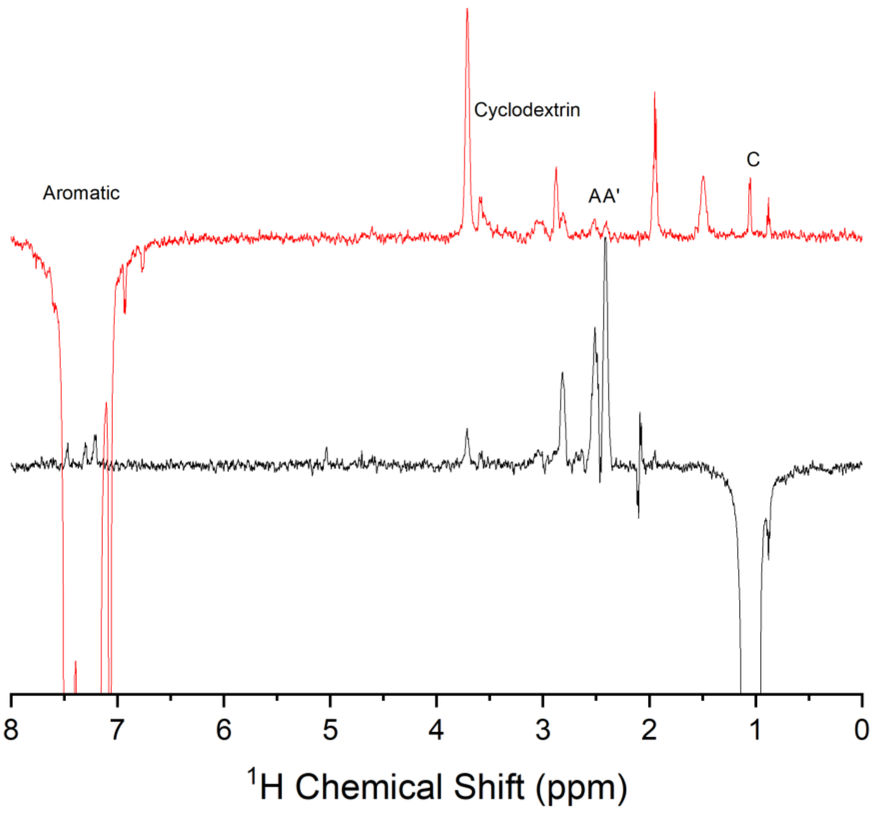

Figure S2. 1D ROESY spectra with irradiation of the fentanyl aromatic protons (top) and the SBX-Me methyl signature (bottom). Irradiated resonances are negative, while any ROESY correlations are positive. The A,A' and C designations follow the assignments from Figure S5. Resonances between 3-4 ppm correspond to proton resonances in the core of the cyclodextrin.

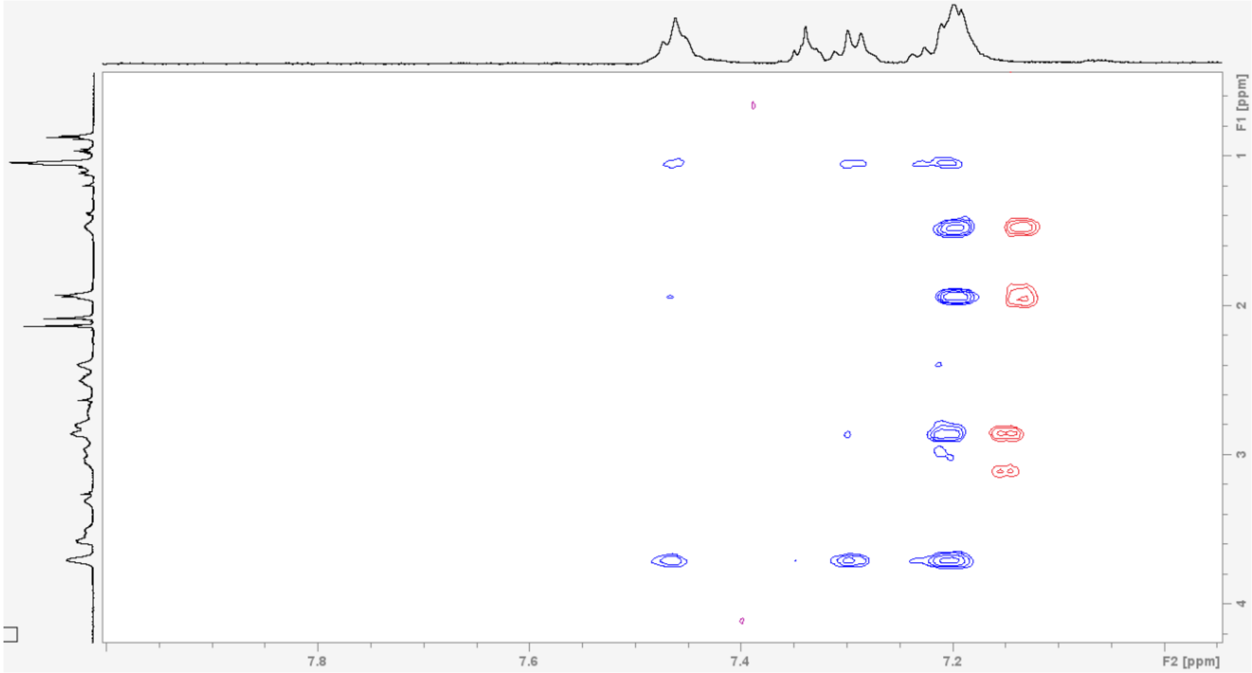

Figure S3. 2D ROESY of the fentanyl+SBX-Me (blue) and the fentanyl (red). The fentanyl spectra is shifted 0.1ppm along the F1 axis for clarity. Correlations between the fentanyl chemical shifts are observable in the pure fentanyl spectra but new correlations at 3.71 and 1.04 ppm correspond to bonding between fentanyl and SBX-Me.

Tissue Distribution Studies for SBX-Me

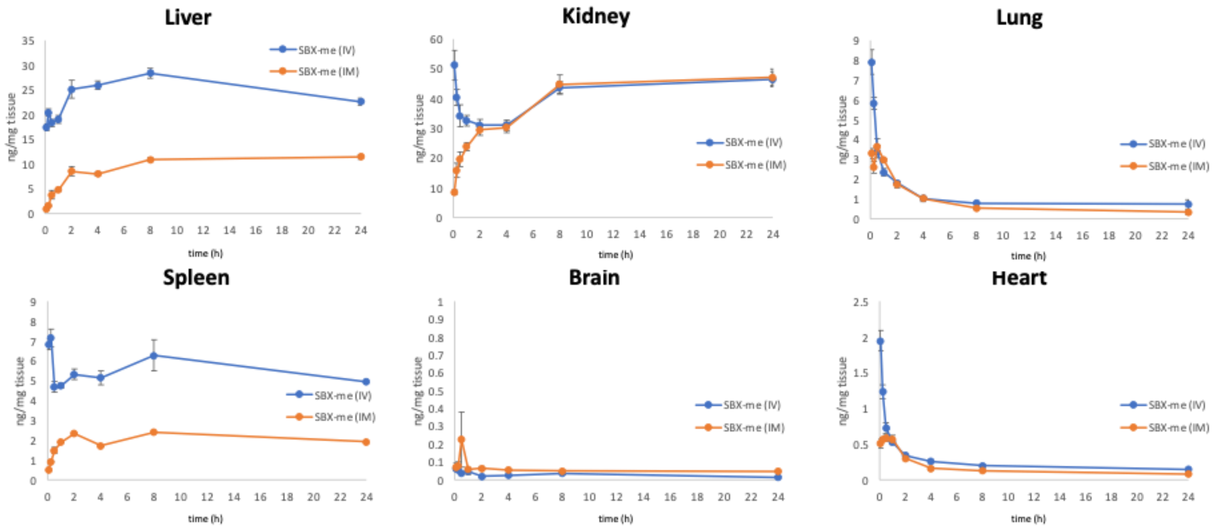

Figure S4. Tissue distribution profiles of SBX-Me following a single intravenous (IV) or intramuscular (IM) administrations of 16 mg/kg ^{14}C-SBX-Me in male Sprague Dawley rats. Data are expressed as the mean of 5 animals ± the standard error.

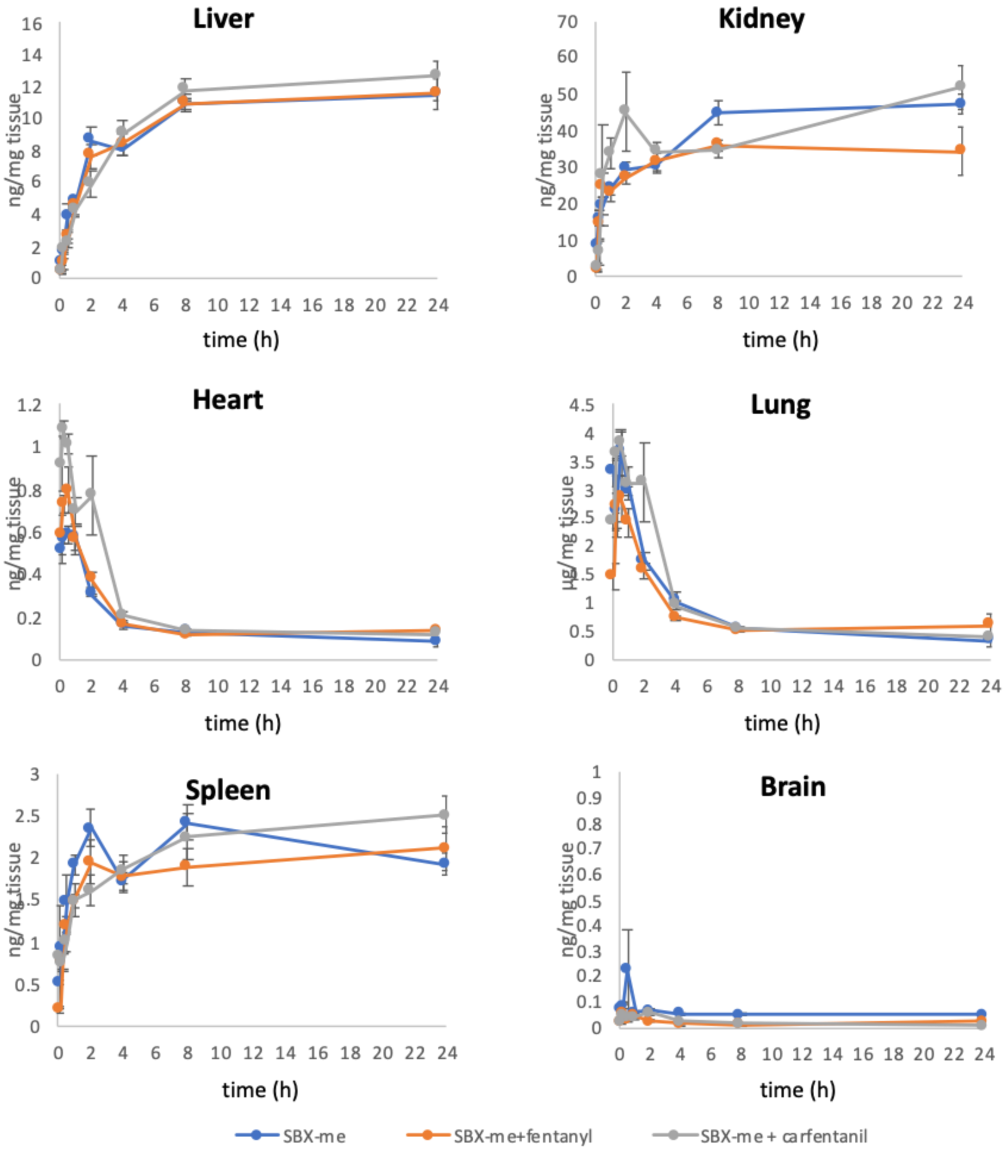

Figure S5. Tissue distribution profiles of SBX-Me following a single (IM) administrations of 16 mg/kg ^{14}C-SBX-Me and an intravenous (IV) administration of 50 mg/kg fentanyl or 5 mg/kg carfentanil in male Sprague Dawley rats. Data are expressed as the mean of 5 animals ± the standard error.

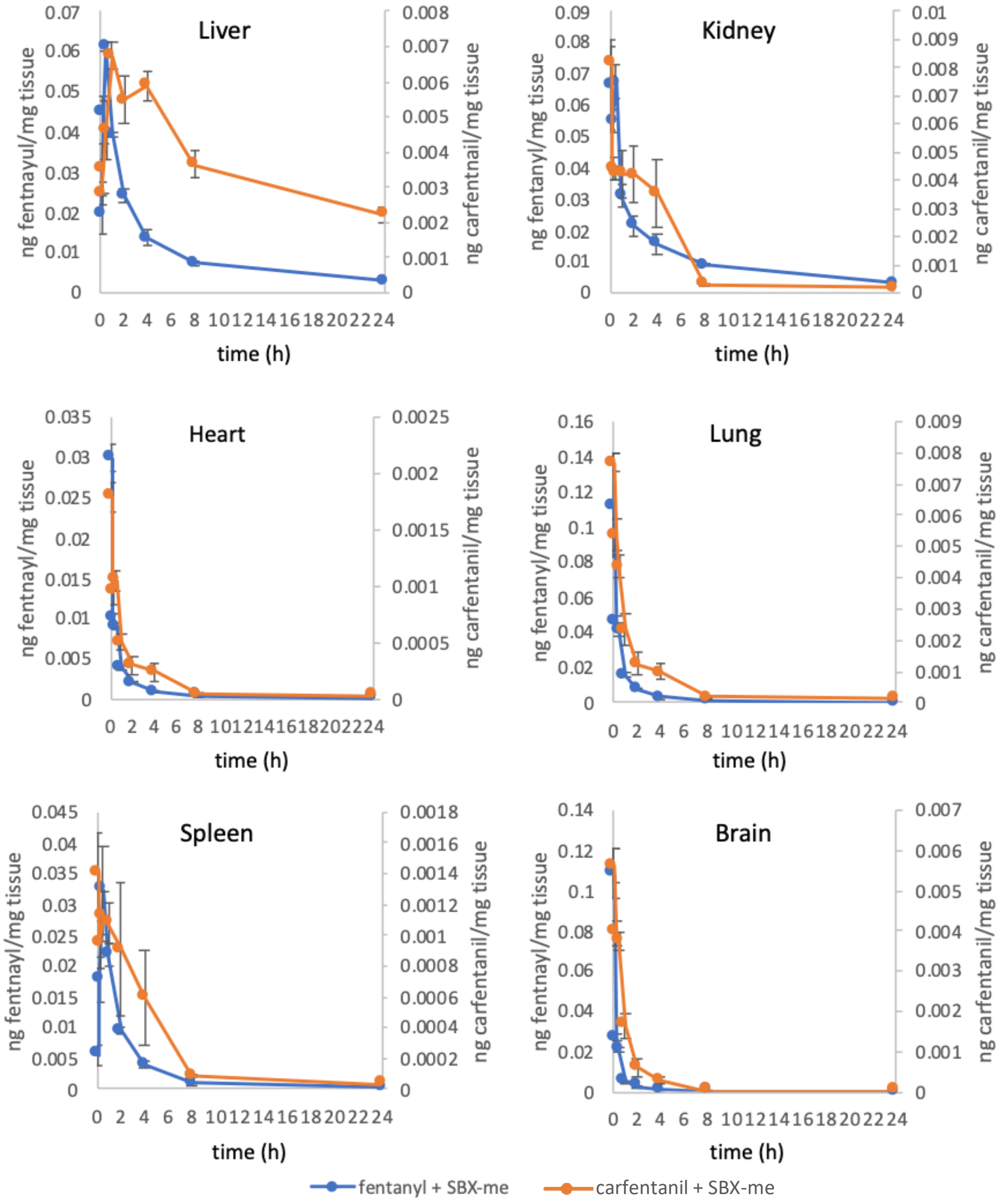

Figure S6. Tissue distribution profiles of fentanyl and carfentanil following single intravenous (IV) administrations of 50 mg/kg ^{14}C-fentanyl or 5 mg/kg ^{14}C-carfentanil and an (IM) administration of 16 mg/kg SBX-Me to male Sprague Dawley rats. Data are expressed as the mean of n=5 animals ± the standard error.

Metabolism. Analysis of the urine of rats exposed to 50 μg/kg ^{14}C-carfentanil or 5 mg/kg ^{14}C-carfentanil and SBX-Me revealed changes in the metabolic profiles of the opioids suggesting the formation of an opioid/SBX-Me complex. Urinary radioprofiles showed a shift in the HPLC retention time of ^{14}C-carfentanil from 8 min to 3-4 min (Figure S4). A similar shift for carfentanil was observed (from 13 min to 3-4 min). The radioactive peaks detected at 3-4 min corresponded to the retention time of ^{14}C-SBX-Me suggesting that the opioids formed a complex with SBX-Me *in vivo* and were excreted in the urine with a retention time similar to SBX-Me. Lastly, another similar shift for remifentanil was observed (from 14 min to 3-4 min). The radioactive peaks detected at 3-4 min corresponded to the retention time of ^{14}C-SBX-Me suggesting that remifentanil, as well as the other opioids formed a complex with SBX-Me *in vivo* and were excreted in the urine with a retention time similar to SBX-Me.

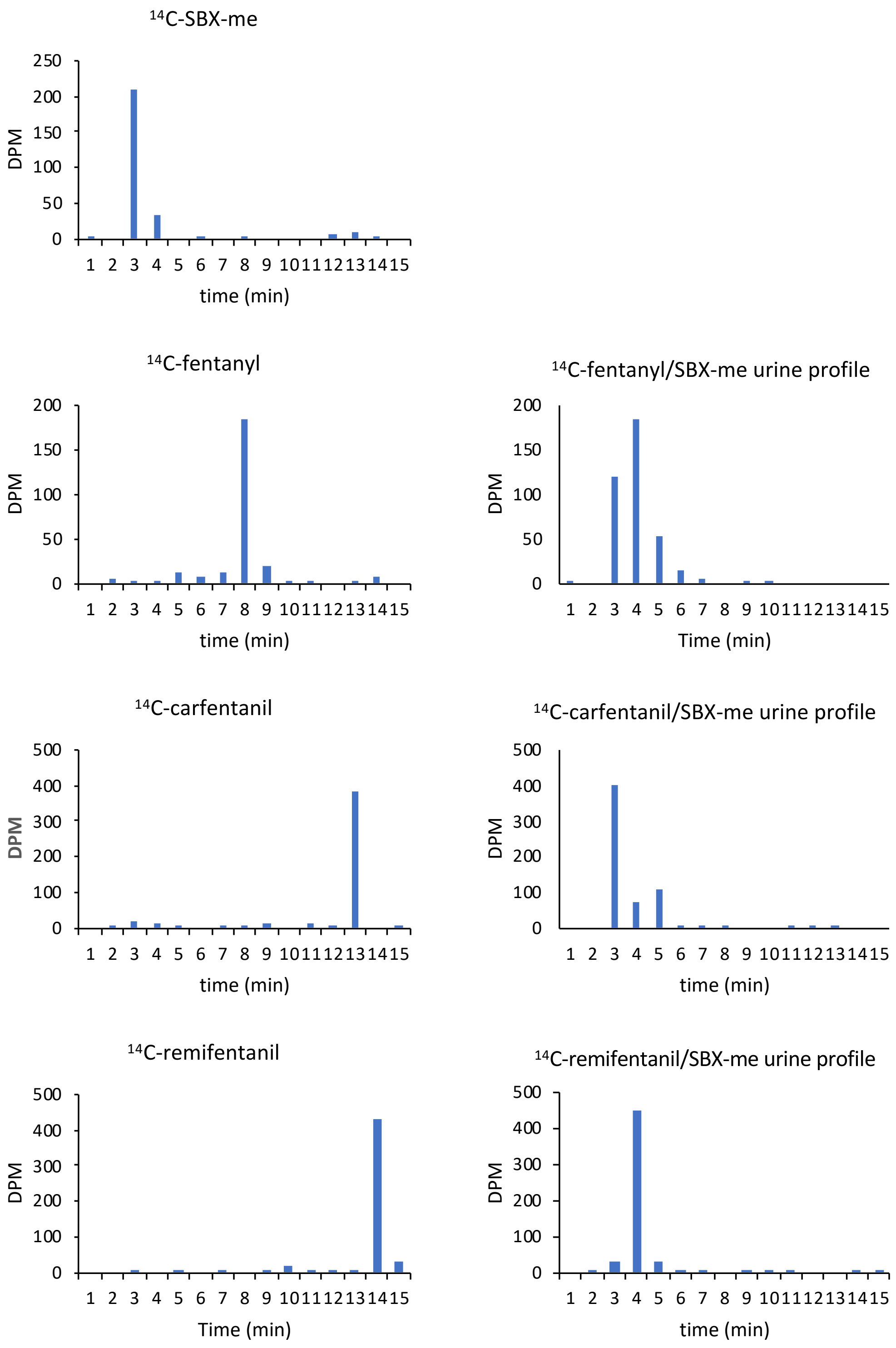

Figure S7. Urinary metabolic radioprofiles of ^{14}C-SBX-Me (16 mg/kg), ^{14}C-fentanyl (50 mg/kg), ^{14}C-carfentanil (5 µg/kg) and ^{14}C-remifentanil (5 mg/kg) following single intravenous (IV) administrations, and urinary metabolic radioprofiles of ^{14}C-fentanyl (50 mg/kg), ^{14}C-carfentanil (5 µg/kg) and ^{14}C-remifentanil (5 mg/kg) from single intravenous (IV) administrations of opioid followed by (IM) administrations of SBX-Me 16 mg/kg to male Sprague Dawley rats.

References

[1] Adam, J. M., Bennett, D. J., Bom, A., Clark, J. K., Feilden, H., Hutchinson, E. J., Palin, R., Prosser, A., Rees, D. C., Rosair, G. M., Stevenson, D., Tarver, G. J., Zhang, M-Q. Cyclodextrin-derived host molecules as reversal agents for the neuromuscular blocker rocuronium bromide: Synthesis and structure-activity relationships. *J. Med. Chem.* **2002**, *45*, 1806-1816.

[2] Mayer, B. P., Kennedy, D. J., Lau, E. Y., Valdez, C. A. Evaluation of polyanionic cyclodextrins as high affinity binding scaffolds for fentanyl. *Sci. Rep.* **2023**, *13*, 2680.
